# Judging AI-generated paintings: art-education differences in source classification

**DOI:** 10.3389/fpsyg.2026.1903106

**Published:** 2026-10-01

**Authors:** Yi Huang, Qiyue Shen, Feng Gan

**Affiliations:** School of Art, Southeast University, Nanjing, China

**Keywords:** aesthetic judgment, AI-generated painting, art-education background, source classification, visual judgment cues

## Abstract

**Introduction:**

Recent advances in generative image models have increased the visual plausibility of AI-generated paintings, raising questions about how viewers attribute image source and whether source-classification performance varies with art-education background. This study examined stimulus-specific patterns of AI/human source classification, differences associated with art education, and participants’ self-reported visual judgment cues.

**Methods:**

A total of 356 participants completed a pairwise source-classification task involving six matched pairs of documented human-made paintings and AI-generated counterparts. Participant-level and stimulus-level classification accuracy were evaluated against the 0.50 chance level, and differences among three art-education groups were examined using Welch’s ANOVA and Holm-adjusted pairwise comparisons. Self-reported visual judgment cues were analyzed among 245 participants who provided complete cue rankings. A supplementary eye-tracking component involving 60 questionnaire participants provided archived group-level heatmaps as qualitative visual context only.

**Results:**

Overall participant-level classification accuracy was modestly but significantly above chance (*M* = 0.549, 95% CI [0.525, 0.573]), *t*(355) = 4.081, *p* < 0.001, Cohen’s *d* = 0.216). However, performance varied substantially across stimuli. Pairs 1, 3, and 6 were classified significantly above chance, Pair 4 did not differ from chance, and Pairs 2 and 5 were significantly below chance. For Pairs 2 and 5, 60.1% and 67.4% of participants, respectively, selected the human-made painting as AI-generated, indicating systematic reversal of source attribution. Art-education background was significantly associated with classification performance, Welch’s *F*(2, 128.292) = 10.135, *p* < 0.001, but only the some-art-background group performed significantly above chance. Among participants with valid cue rankings, facial and hand details were commonly assigned higher priority, whereas background details were most frequently ranked last; neither first- nor last-ranked cue distributions differed significantly across art-education groups.

**Discussion:**

AI/human painting source classification was characterized by marked stimulus-specific directionality rather than a uniform level of discrimination difficulty. The below-chance performance observed for two stimulus pairs indicates systematic source-attribution reversal, while the art-education results do not support a simple monotonic expertise advantage. These findings suggest that source judgments depend on interactions among stimulus-specific visual properties, viewers’ art-education backgrounds, and reported judgment strategies rather than on a single general ability to distinguish AI-generated from human-made paintings.

## 1 Introduction

Recent advances in generative image models have substantially changed the production and reception of visual art. AI-generated paintings can now imitate historical styles, reconstruct figure-based images, and produce visually coherent works that are difficult for many viewers to distinguish from human-made paintings. This development raises an empirical question concerning how viewers judge the creative source of paintings and whether such judgments vary with art-education background.

This study employs source classification based on questionnaire surveys as its primary empirical method. Specifically, the study examined how participants with different backgrounds in art education classified the source of six matched sets of human-made paintings and AI-generated counterparts, as well as which visual details they retrospectively reported when making these judgments. In addition, a supplementary eye-tracking experiment was conducted on a smaller subsample. This component was designed from the outset as a supplementary observational reference, rather than as a second experiment or an independent test of visual attention mechanisms.

The definition of AI painting can be summarized as follows: based on computer intelligence technology, humans use machine language to imitate and learn painting art, thereby developing autonomous, independent, and creative painting skills and producing original works. Within this concept, artificial intelligence is regarded as an auxiliary tool for artistic creation, attempting to express unique artistic styles through simulation and learning. From the 1956 Dartmouth Conference to the present day, the development of AI-generated painting has been inextricably linked to rapid technological advancements (Moor, 2006; McCarthy et al., 2006). Initially, AI garnered attention primarily through mimicking human intellect. However, with the continuous advancement of deep learning technology, machines have gradually attained higher levels of autonomy and the ability to process multifaceted information. This has opened broader possibilities for AI integration into the field of painting (Liu, 2020). In artistic creation, a key focus is whether the complex processes experienced by human artists, such as emotions and thought, can be fully simulated by AI. Some argue that artistic creation requires the subjective involvement of the artist, viewing AI merely as a tool (Hong and Curran, 2019). Yet, as technology advances, AI has progressed beyond replicating artists’ works to assisting in creation and even producing independent pieces, prompting a reexamination of subjectivity in artistic creation. Kant defined art as “the free play of the mind,” where the creative process itself is an end, bringing pleasure to the subject (Kant, 1987). Fechner posited that artists encode emotions within formal structures, while audiences decode these emotions through perceiving the structure, creating a bidirectional esthetic exchange (Fechner, 1876). Therefore, evaluating AI-generated paintings requires considering two elements: the creator’s free will and the work’s esthetic value in reflecting the objective world. Gestalt theory provides a general conceptual framework for considering how local pictorial details are perceived in relation to an organized visual whole. In figure-based paintings, facial features, hands, clothing, and surrounding pictorial elements may contribute differently to viewers’ judgments of image coherence. In the present study, however, these relationships were not tested through controlled part–whole manipulations or quantitative AOI-based eye-tracking measures. Gestalt principles are therefore used only as an interpretive framework for discussing questionnaire responses and descriptive heatmap patterns, rather than as an empirically tested perceptual mechanism.

## 2 Literature review and research

### 2.1 Technical development of AI painting

AI-generated art, as a critical branch of generative artificial intelligence (Generative AI), is redefining the technological paradigms and evaluative frameworks of artistic creation. By transforming visual art production into computationally analyzable and iteratively optimizable data processes through algorithmic models, this field signifies a paradigm shift from anthropocentric creation to human-machine symbiosis (Tzafestas, 2016). The interplay between AI and painting traces its origins to mid-20th century computational experiments generating basic geometric patterns. Emerging from the foundational 1956 conceptualization of artificial intelligence—defined as systems replicating human cognitive capacities—the initial phase of “computer-generated art” established critical intersections between artistic practice and computational evolution. Pioneering works like Benjamin F. Laposky’s 1952 Oscillon series demonstrated algorithmic pattern generation, though these early outputs remained artist-driven rather than collaborative (Edler, 2020). The developmental trajectory entered its formative AI-art phase through seminal institutions like the Slade School of Fine Art’s Experimental and Computing Department at University College London. Harold Cohen’s AARON system (1973) epitomized this era, employing rule-based algorithms to simulate organic brushstroke dynamics—a milestone in procedural esthetic mimicry (Sundararajan, 2014).

The 1980s witnessed pivotal advancements in machine learning that catalyzed a paradigm shift in computational art creation. This period saw neural network innovations enabling broader applications of artificial intelligence in artistic domains, particularly through enhanced pattern recognition and procedural automation. Subsequent exponential growth in computing power during the 1990s provided critical infrastructure for developing sophisticated AI painting systems, while breakthroughs in image recognition and data mining techniques expanded cross-disciplinary applications in architecture and music composition.

Ramachandran and Hirstein’s neuroaesthetic framework posits that artistic essence manifests through peak shift effects—perceptual exaggerations of essential features creating hyper-stimuli that transcend literal representation (Ramachandran and Hirstein, 1999). Contemporary progress accelerated through foundational work by Geoffrey Hinton, whose pre-2006 contributions to deep learning architectures laid essential groundwork. A watershed moment occurred in 2014 with Ian Goodfellow’s introduction of Generative Adversarial Networks (GANs), comprising generator-discriminator dyads that optimize through adversarial training to produce photorealistic outputs (Goodfellow et al., 2014). Subsequent innovations like Editing GANs and Creative Adversarial Networks further expanded creative synthesis capabilities.

The post-2015 era witnessed unprecedented technical refinement:

- Karras et al. (2020) achieved record-breaking 1024 × 1024 facial generation via style-modulated generators on FFHQ datasets.
- Ho et al. (2020) developed Denoising Diffusion Probabilistic Models (DDPMs), which advanced generative image modeling through a stochastic denoising framework.
- Radford et al. (2021) introduced the CLIP architecture, establishing transformer-based visual encoding that became critical to later text-to-image breakthroughs.
- Rombach et al. (2022) optimized computational efficiency through latent space diffusion, reducing the computational cost of high-resolution image synthesis.

In addressing the technical challenges posed by diffusion models in artistic style transfer, Sun and Meng (2025) proposed StyDiff, a framework that combines adaptive instance normalization (AdaIN) with the diffusion process. By reducing over-stylization while improving the preservation of content structure, this framework highlights a persistent limitation of current generative models: the difficulty of maintaining consistency in local details. This limitation offers a technical explanation for the hand-detail distortions observed in the AI-generated portrait materials used in the present study.

Complementing technical advances, Iigaya et al. (2021) demonstrated through computer vision analysis that human esthetic preferences arise from hybrid processing of low-level features, such as tonal contrast and spatial composition, and high-level semantic attributes, such as emotional valence and conceptual specificity.

### 2.2 Human experience and algorithmic image generation

From the perspective of cognitive psychology, Bianchi et al. (2025) synthesize research on the esthetic evaluation of AI-generated art and argue that viewers’ responses to such works are shaped by multiple factors, including expectations about style, processing fluency, and beliefs about creativity. This framework provides a more nuanced cognitive-psychological explanation for the significant differences observed in the present study among participants with different art-education backgrounds when distinguishing AI-generated paintings from human-made paintings.

Under more tightly controlled experimental conditions, van Hees et al. (2025) used paired presentations to reduce the influence of explicit authorship labels. They found that human viewers could still distinguish AI-generated paintings from human-made paintings at above-chance levels even when no label information was available. At the same time, the study reported a significant esthetic preference for AI-generated artworks. This paradoxical pattern—being able to discriminate AI-generated art while also preferring it—suggests that perceptual identification and esthetic evaluation do not follow a simple linear relationship but are regulated by partly different cognitive mechanisms.

Research on abstract painting also indicates that esthetic experience is closely related to perceptual contrast and statistical properties of visual composition, which further supports the claim that viewers’ responses to AI-generated painting should be discussed not only in terms of authorship but also through measurable perceptual features (Mallon et al., 2014).

Prior research provides several lines of context for the present empirical question. First, generative image models have increased the capacity of AI-generated painting for stylistic imitation and visual reconstruction, while local structural consistency may still remain a perceptible limitation. Second, studies on viewers’ classification, preference, and authorship judgments show that responses to AI-generated art are unstable and may be influenced by labels, familiarity, esthetic expectations, and beliefs about creativity. Third, art-education background may be associated with differences in judgment strategies, while previous visual-attention research provides a broader context for considering how viewers inspect pictorial information. Expertise, however, does not necessarily translate into a simple linear improvement in AI/human source classification. The following review is therefore organized around AI-generated image realism, human-AI art discrimination, visual attention, and expertise-related differences in esthetic judgment.

The use of AI in fine art has changed significantly with the commercial success of AI artifacts, open-source projects, and continued advances in AI technology. The development of these technologies has provided artists with new creative tools, while also provoking reflection on traditional ways of creating art. The intervention of AI has triggered a revolutionary change in the art field, with more and more artists combining algorithms with creative concepts. However, with the wide application of AI in the art field, a series of problems and challenges has emerged. Issues such as the attribution of creative rights, originality and uniqueness, evaluation standards of works, and the role of AI in the creation of art paintings need to be studied and discussed in depth. Consideration from legal, ethical, and economic perspectives is required to promote the healthy development of AI in the field of art. In general, the application of AI in the field of art presents a rich development trend, providing artists with new creative tools and ideas. However, the ensuing problems and challenges also require in-depth thinking and solutions to promote the constructive interaction between AI and art.

In the present study, AI-generated painting refers to a visual image generated using an artificial-intelligence image-generation model in response to human-provided textual prompts and/or source images. The term concerns the production method of the experimental image and does not imply autonomous artistic agency on the part of the AI system.

## 3 Methods

The present study was designed primarily as a questionnaire-based empirical investigation of AI/human painting source classification. Participants completed six pairwise classification items, each consisting of a documented human-made source painting and an AI-generated counterpart. Participant-level classification accuracy was calculated as the proportion of correctly identified AI-generated images across the six matched pairs. The primary quantitative evidence therefore consists of questionnaire-based source-classification accuracy and self-reported visual judgment cues.

A smaller eye-tracking session was conducted as an ancillary observational component. It was not designed as a second experiment equivalent to the questionnaire study and was not included in the primary inferential analysis. See Section 3.5 for details.

The study involved 356 participants in the primary questionnaire analysis. Of these participants, 60 subsequently participated in the supplementary eye-tracking session, with 20 participants drawn from each of the three art-education groups. Thus, the questionnaire cohort and the eye-tracking group were not independent samples; the latter constituted a subsample of the former. It should be noted that the heatmap records gaze data obtained when participants, having already completed the discrimination task and understood the experimental procedure, view the familiar stimuli again.

The two components nevertheless provide different levels of evidence. Participant-level questionnaire responses were retained and used for statistical analyses of source-classification performance and self-reported judgment cues. In contrast, the retained eye-tracking outputs consisted only of aggregated group-level heatmaps rather than participant-level fixation records (see Section3.5).

### 3.1 Questionnaire participants and procedure

In March 2025, the questionnaire A Comparative Study of AI-Generated Painting and Human Painting was distributed through the Wenjuanxing platform. A total of 358 questionnaires were distributed, of which 356 valid responses were recovered and analysed using SPSS.

Participants younger than 18 years were included only when written informed consent had been obtained from a parent or legal guardian. Age-appropriate assent was also obtained from the minor participants prior to participation. The study protocol, including the recruitment of minors, was reviewed and approved by the Institute for the Evaluation of Art Development at Southeast University.

The questionnaire collected demographic information, familiarity with AI-generated painting, attitudes toward AI-generated painting, esthetic preferences, and responses to six pairwise source-classification items. In each item, participants viewed a documented human-made source painting together with its AI-generated counterpart and selected the image that they believed had been generated by AI. The resulting accuracy measure therefore represents pairwise source-classification performance within the present stimulus set rather than general AI-art detection or recognition of modified originals.

These quantitative questionnaire data are used to characterize participants’ esthetic attitudes, esthetic preferences, and performance in classifying the creative source of the images.

In addition to the source-classification items, the questionnaire included a self-reported visual judgment-cue task. This was a forced-ranking task rather than a free-text or multiple-selection question. Participants were presented with four predefined categories of pictorial detail—facial details, hand details, clothing details, and background details—and were asked to rank all four categories according to the order in which they believed they attended to or used these details when judging whether an image was AI-generated or human-made, from the first to the last.

Each category could occupy only one rank position, producing a complete ordinal sequence across the four predefined cue categories. A response was treated as valid only when all four categories were ranked once and a complete ordering was provided. Responses that were incomplete or did not conform to the ranking instructions were excluded from the descriptive cue analysis.

The ranking data were analysed descriptively. We report the frequencies of the most common complete ranking sequences and the number and percentage of valid respondents who placed each cue category in the first or last position. Because this was a single retrospective ranking task rather than a multi-item psychometric scale, internal-consistency reliability was not estimated. No test–retest reliability data were collected. The results should therefore be interpreted as descriptive self-reports of judgment strategy rather than as objective measures of visual-attention sequence.

### 3.2 Stimuli and apparatus

Six documented human-made paintings were selected as the source works for the matched stimulus pairs. The stimulus bank includes single portraits, double-figure paintings, and narrative scenes with human figures, which are collectively categorized as figure-oriented and thematically relevant painting samples in this paper. Stable Diffusion 3.5 was used to generate the AI counterparts because it supports high-resolution text-guided and image-to-image generation. This model possesses robust high-resolution image synthesis capability and achieves outstanding prompt adherence. The AI images were generated strictly following the thematic content, integral composition, figure layout and stylistic traits of the original paintings to acquire similar visual effects without being identical to the source works (Figures 1–6).

**Figure 1:**
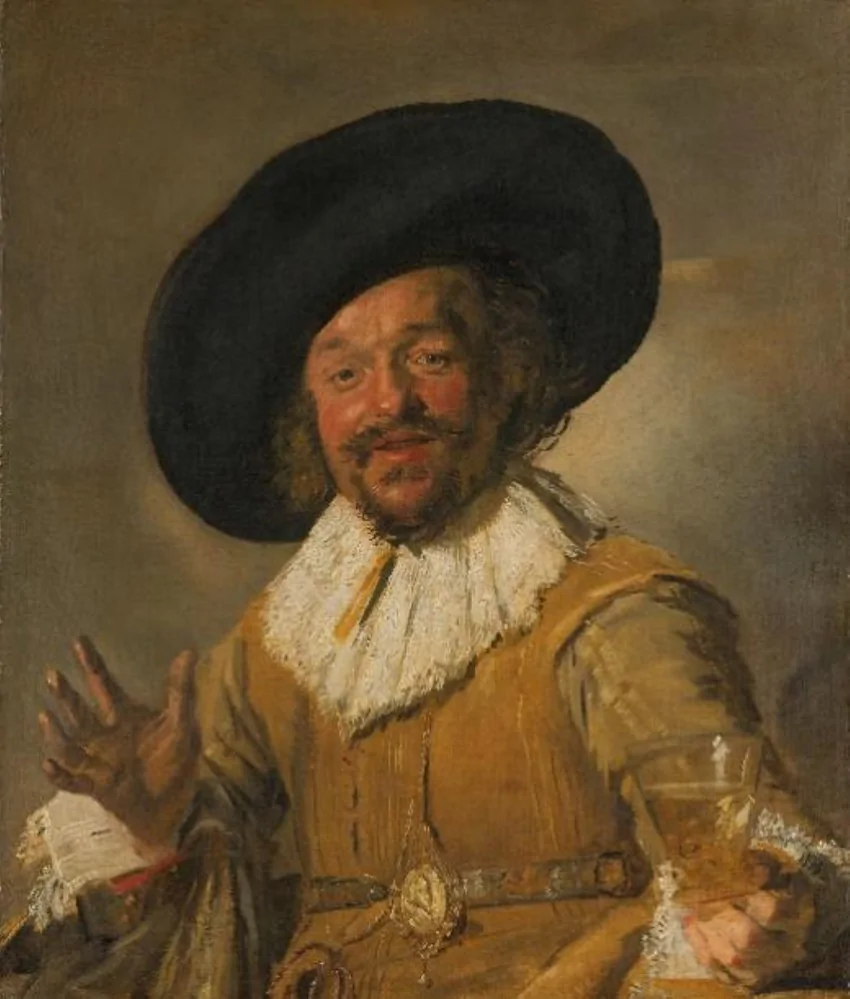
Frans Hals, The Merry Drinker (c. 1628–1630) (reproduced with permission from the Rijksmuseum, Amsterdam, object no. SK-A-135, https://www.rijksmuseum.nl/en/collection/object/The-Merry-Drinker--884b50ad9157e0b12a90e4a5e8acf336?tab=data, licensed under CC0).

**Figure 2:**
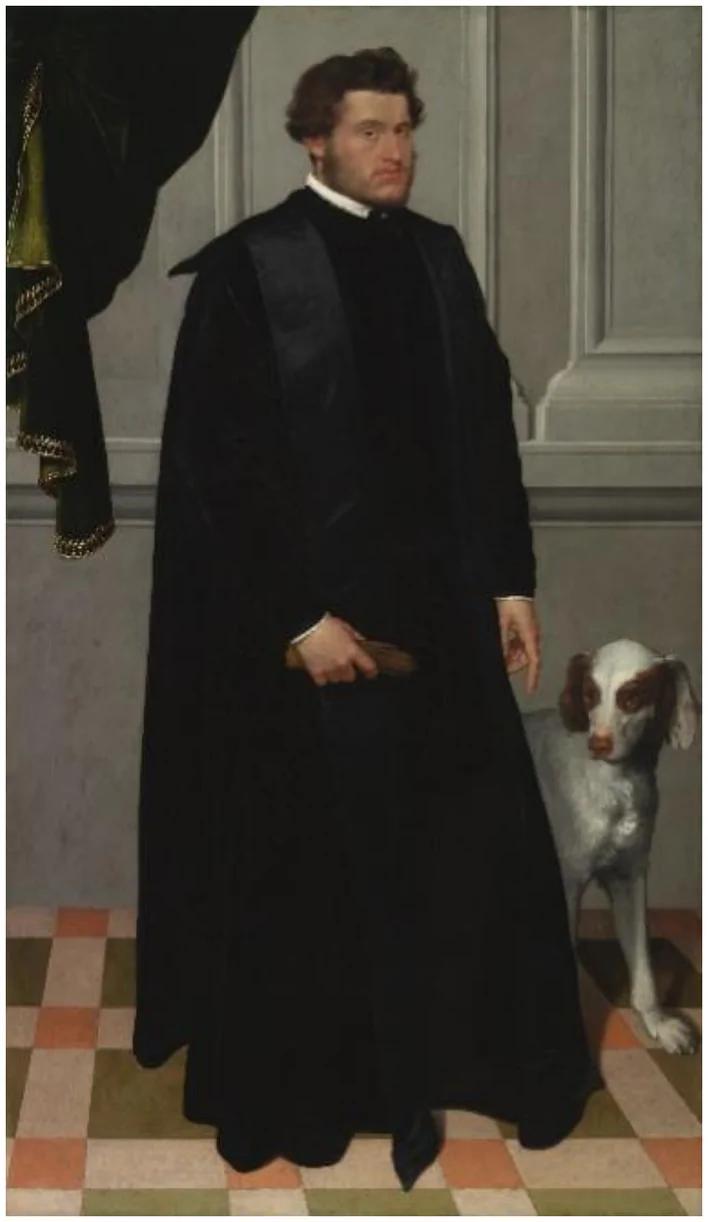
Giovanni Battista Moroni, Gian Lodovico Madruzzo (1551–1552) (reproduced with permission from The Art Institute of Chicago, reference no. 1929.912, https://www.artic.edu/artworks/4081/gian-lodovico-madruzzo, licensed under CC0).

**Figure 3:**
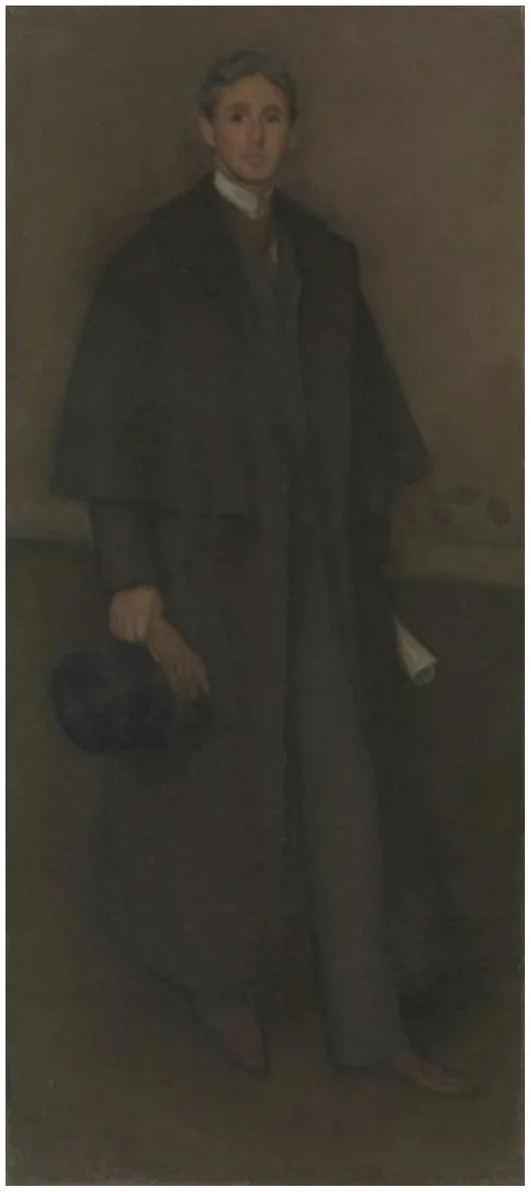
James McNeill Whistler, Arrangement in Flesh Color and Brown: Portrait of Arthur Jerome Eddy (1894) (reproduced with permission from The Art Institute of Chicago, reference no. 1931.501, https://www.artic.edu/artworks/8958/arrangement-in-flesh-color-and-brown-portrait-of-arthur-jerome-eddy, licensed under CC0).

**Figure 4:**
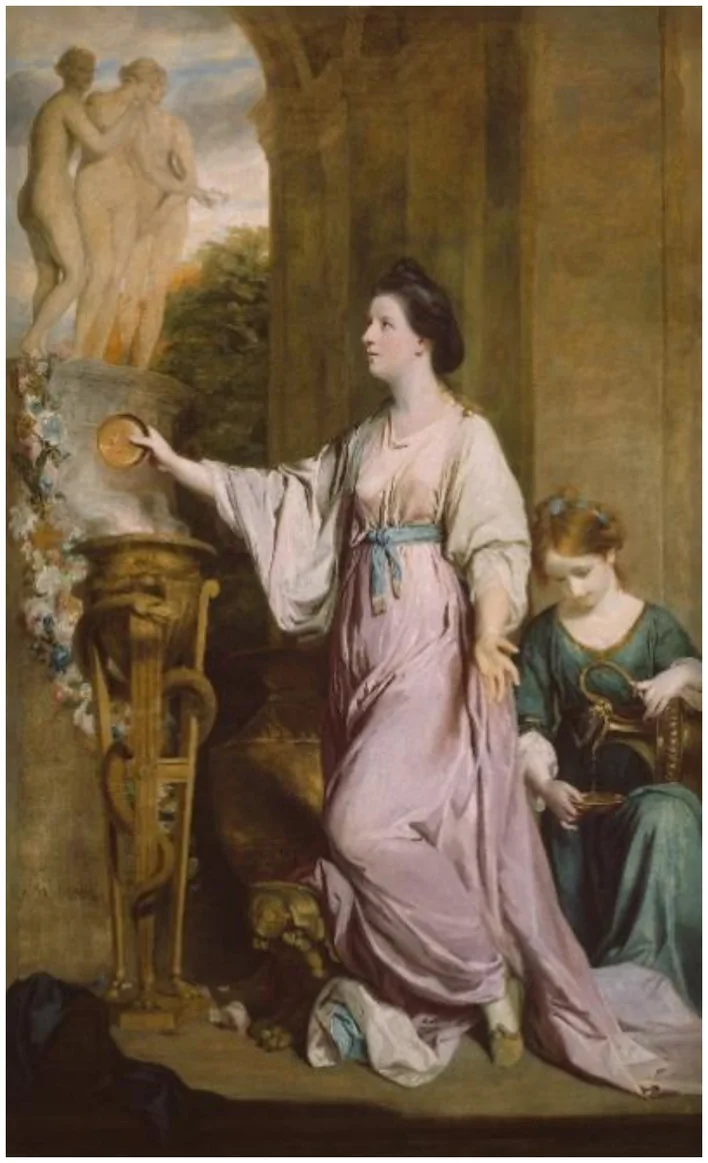
Sir Joshua Reynolds, Lady Sarah Bunbury Sacrificing to the Graces (1763–1765) (reproduced with permission from The Art Institute of Chicago, reference no. 1922.4468, https://www.artic.edu/artworks/4788/lady-sarah-bunbury-sacrificing-to-the-graces, licensed under CC0).

**Figure 5:**
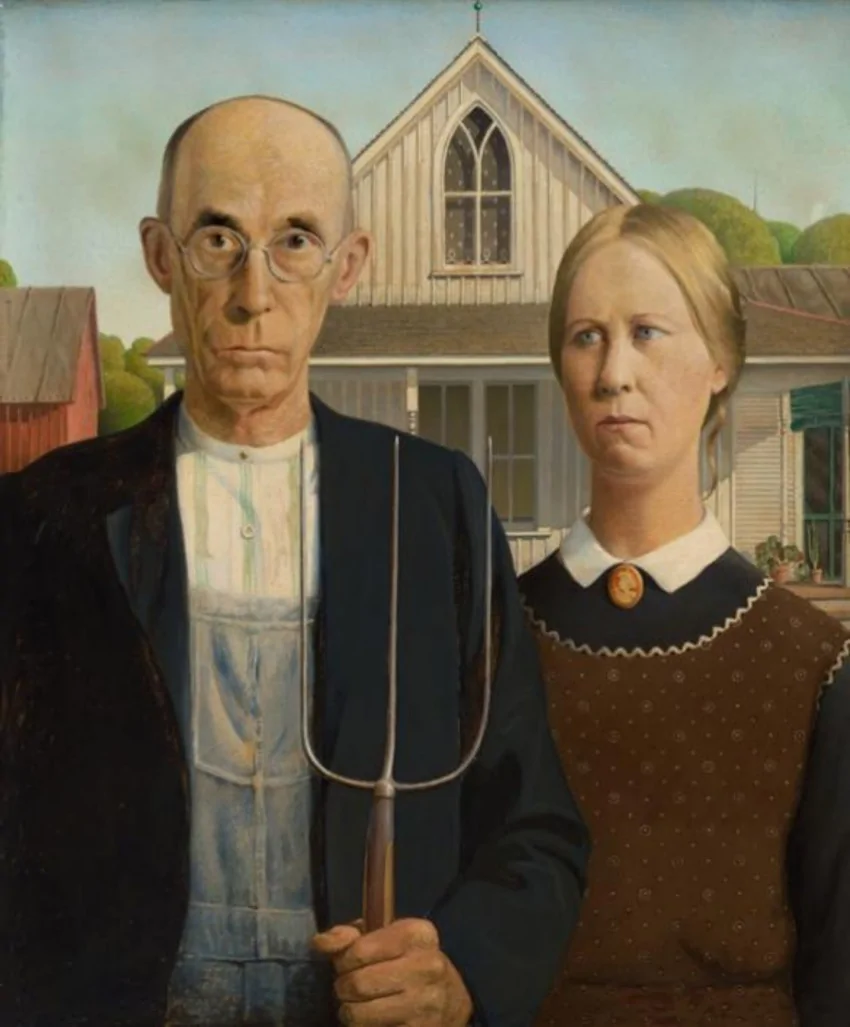
Grant Wood, American Gothic (1930) (reproduced with permission from The Art Institute of Chicago and the Friends of America Art Collection, reference no. 1930.934, https://www.artic.edu/artworks/6565/american-gothic).

**Figure 6:**
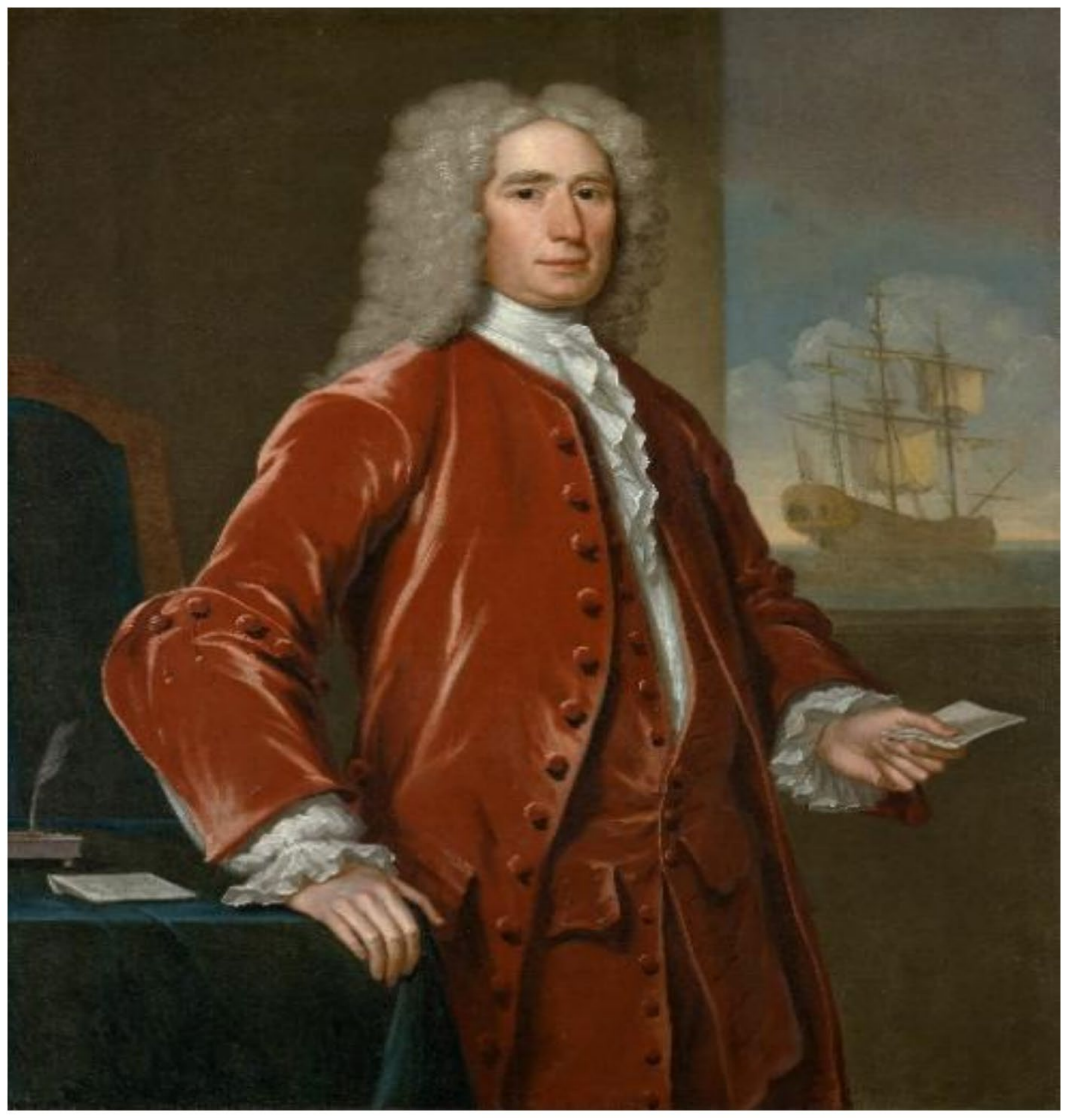
John Smibert, Richard Bill (1733) (reproduced with permission from The Art Institute of Chicago, reference no. 1944.28, https://www.artic.edu/artworks/49702/richard-bill, licensed under CC0.

#### 3.2.1 AI-generated experimental materials

Text-guided image synthesis served as the primary generation framework, supplemented by an image-to-image mode that used the original paintings as references. Customized prompts fully captured the core theme, number and arrangement of figures, overall compositional structure, painting style, and various visual features of each original artwork. Complete generation logs were not retained; consequently, not all generation parameters can be reconstructed retrospectively.

During the stimulus preparation phase, multiple candidate AI images were generated for each original painting, and the final matched AI images were selected based on three criteria: first, preserving the core theme and overall compositional structure of the original work; second, maintaining sufficient stylistic and visual comparability with the original to ensure participants could effectively perform the discrimination task between human-made and AI-generated paintings; and third, being free of conspicuous generation artifacts that would allow participants to identify the image source at a glance. The screening of candidate images was completed prior to formal participant testing, and the selection process was independent of subsequent classification accuracy and participant response data.

1. Text-guided Generation

Applied to all six sets of original artworks as the primary pathway for generating baseline candidates:

Each original artwork was first annotated with multi-dimensional textual descriptions to refine precise prompts covering subject matter, compositional structure, painting medium, color tonality, lighting and shadow characteristics, and artistic style. Text-to-image generation was executed using pure text prompts as input. For each original artwork, 2–3 independent generation rounds were run, producing a fixed number of candidate samples per round. Detailed explanation of prompt generation (Supplementary Section 1.3).

1. Image-to-image Generation

Used for optimized generation of experimental stimuli to increase the proportion of high-fidelity stimulus materials:

The corresponding original human painting was used as the reference base image and input into the image-to-image module.

The refined precise prompts from the text-guided phase were adopted, supplemented by constraint terms for image fidelity. Samples balancing the original work’s characteristics and AI generation completeness were produced via the repainting mechanism.

Gradient levels of repainting strength were set to generate candidate samples with low, medium, and high degrees of fidelity to the original, enriching the gradient of visual variation across the stimulus set.

1. Candidate Samples and Screening

This study adopted a multiple-candidate generation strategy, producing multiple independent candidate samples for each original artwork for a total of 120 raw samples. All samples underwent initial manual screening and were evaluated strictly across four dimensions: compositional matching, stylistic similarity, color fidelity, and image completeness. Invalid samples with obvious generation defects or severe deviation from the original work’s characteristics were excluded, ultimately forming a candidate pool of experimental stimuli for subsequent selection and grouping in the formal experiment.

The six final AI-generated paintings are presented in Figures 7–12.

**Figure 7:**
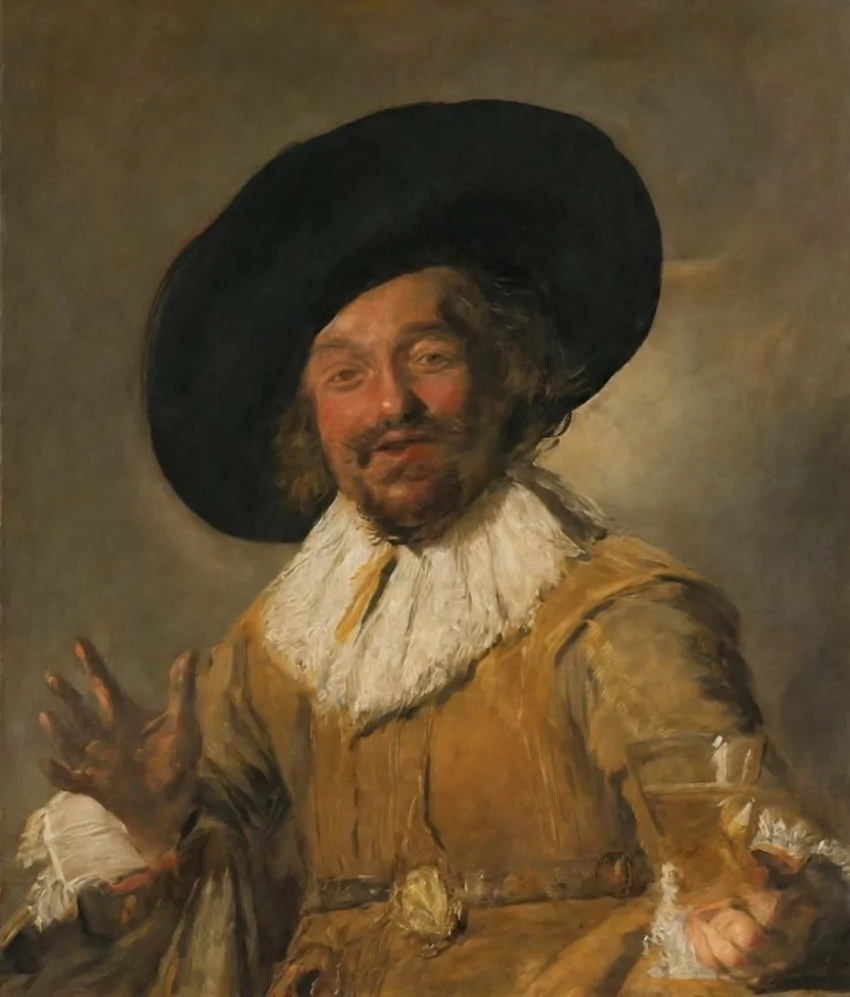
AI Art 01.

**Figure 8:**
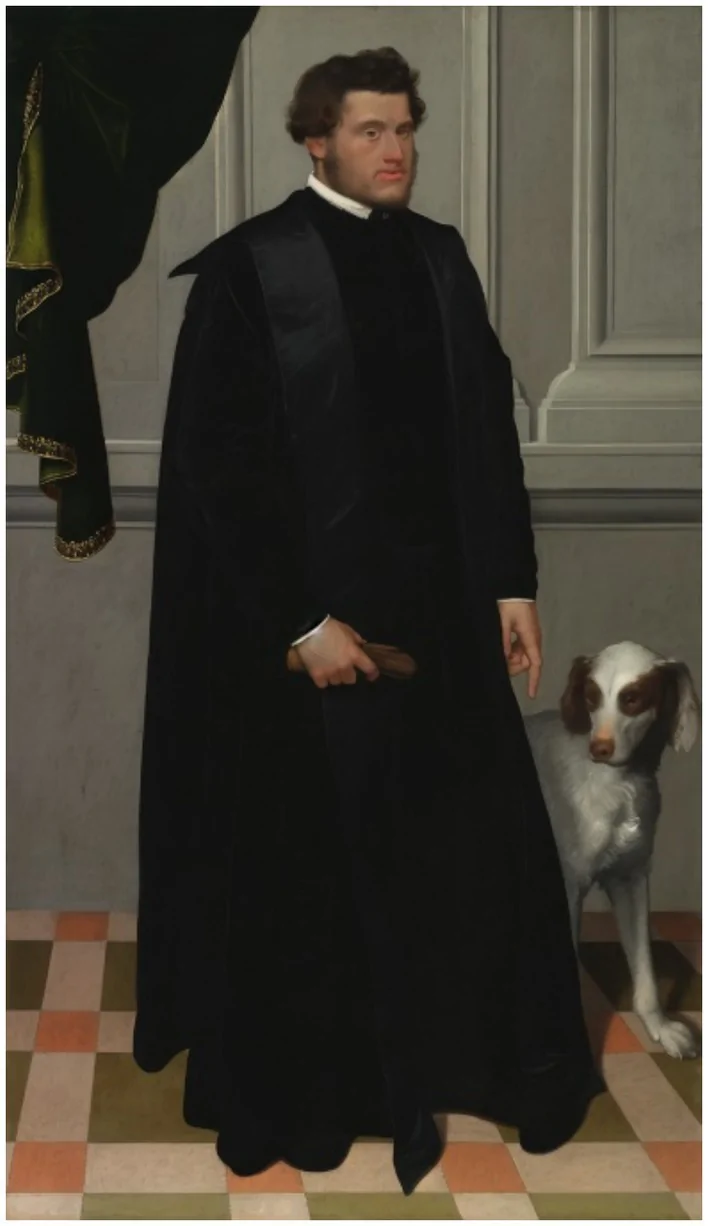
AI art 02.

**Figure 9:**
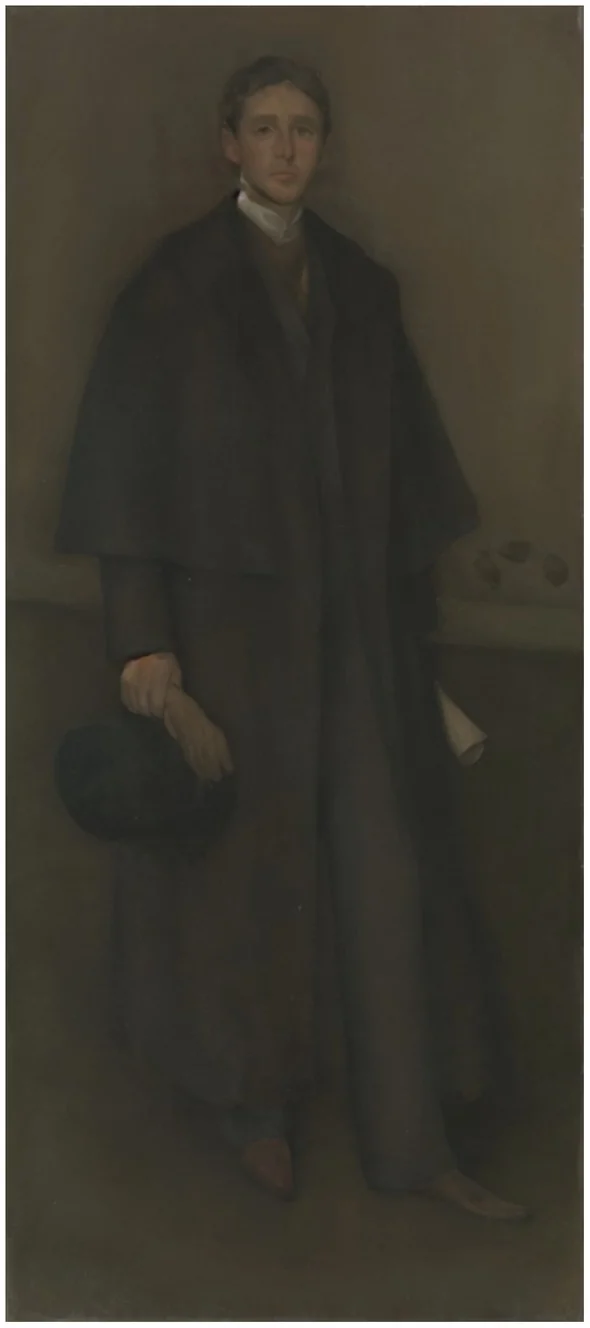
AI Art 03.

**Figure 10:**
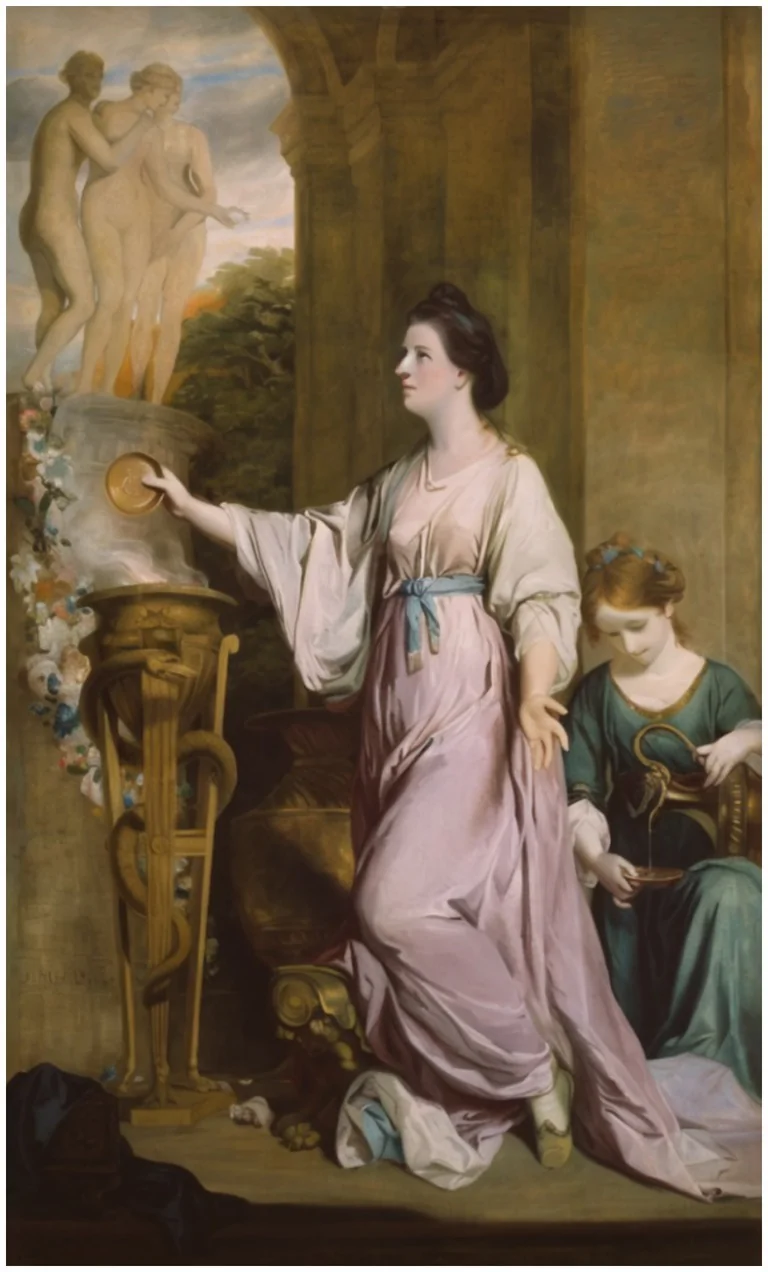
AI art 04.

**Figure 11:**
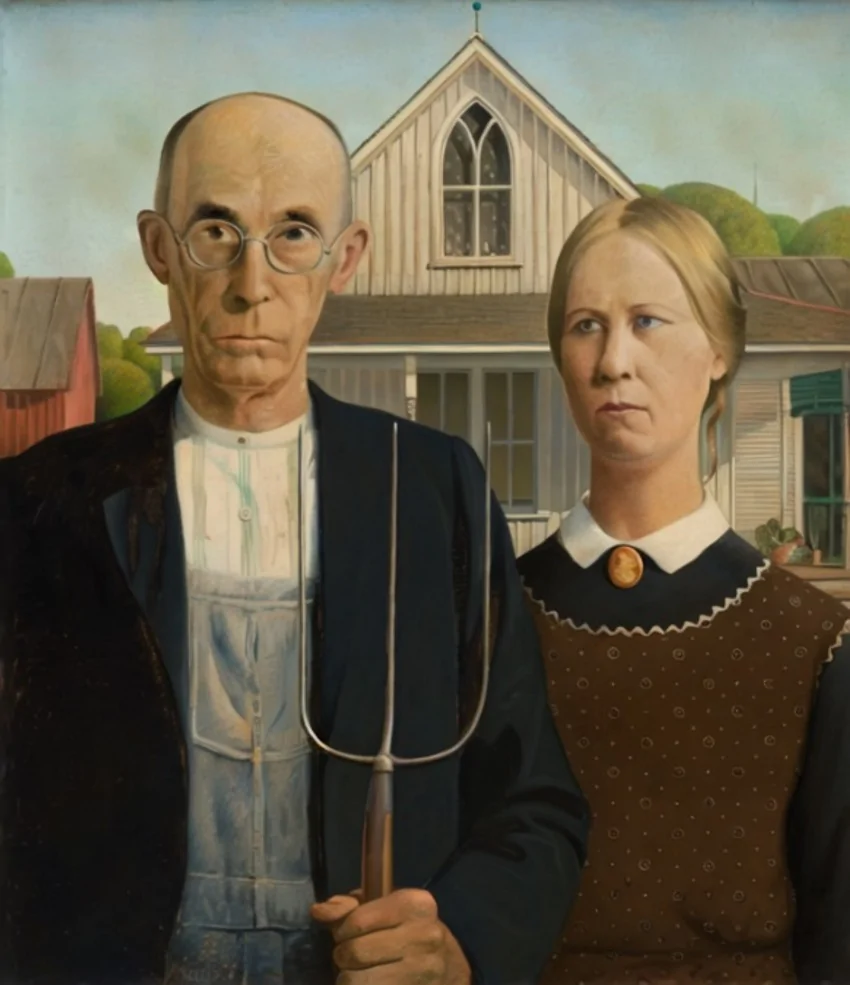
AI Art 05.

**Figure 12:**
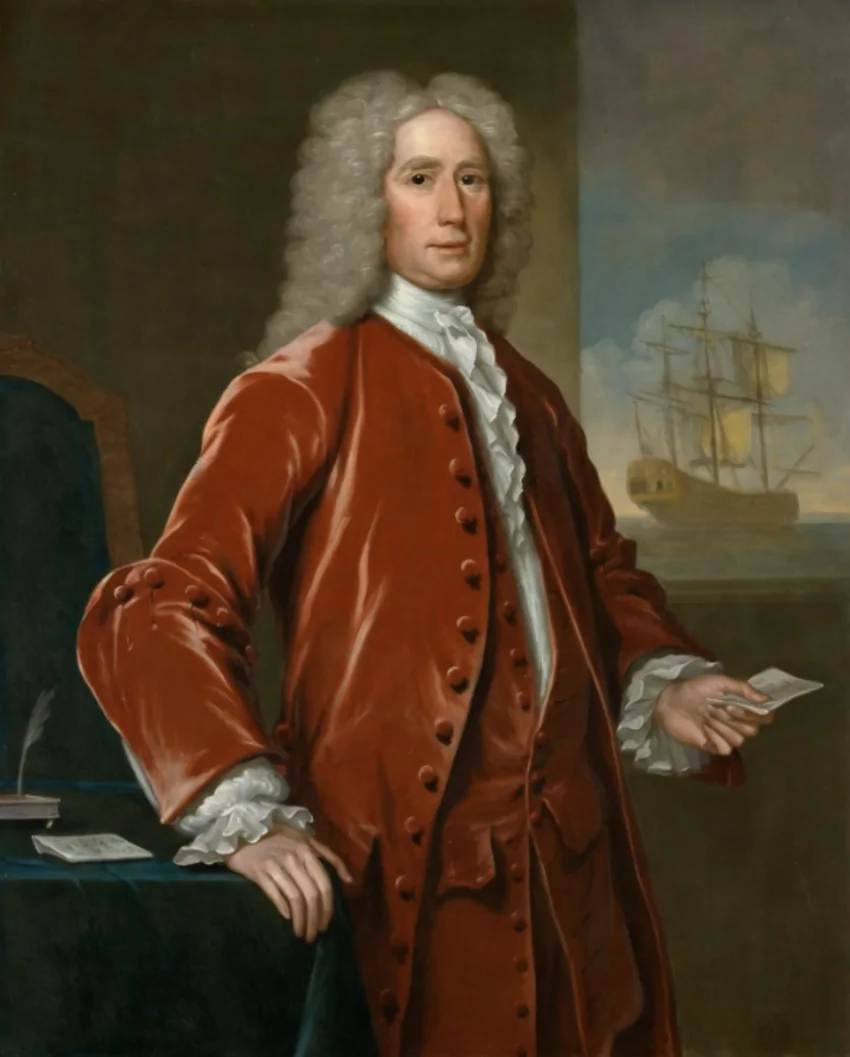
AI Art 06.

#### 3.2.2 Experimental equipment and presentation methods

In the supplementary eye-tracking experiment, visual stimuli were presented on a 60-inch display with a resolution of 1920 × 1080 pixels. The experiment was conducted in a controlled laboratory environment to minimize external distraction. Gaze behavior was recorded using the D-Lab system developed by Ergoneers together with Dikablis eye-tracking glasses. In this study, the retained eye-tracking output data consists of aggregated heatmap visualizations. See Section 3.5 for details.

### 3.3 Procedure

To approximate the viewing conditions of an art gallery, participants viewed the images while standing rather than sitting. Wearing Dikablis eye-tracking glasses, they viewed the AI-generated and human-made paintings from a distance of 100 cm from the screen, with a fixed tabletop placed in front of the body for support (Supplementary Figure S1). The experiment began with the eye-calibration procedure provided by D-Lab (Supplementary Figure S2). Each set of works was viewed for 30 s, followed by a 10-s rest period to reduce eye fatigue (see Section 3.5).

### 3.4 Statistical analysis

Participant-level source-classification accuracy was calculated as the proportion of correctly identified AI-generated images across the six matched pairs. Because each item involved a two-alternative forced-choice decision, random responding corresponds to an expected accuracy of 0.50. Overall participant-level accuracy was therefore compared with 0.50 using a two-sided one-sample *t*-test, with the 95% confidence interval and Cohen’s *d* reported as measures of precision and effect size.

Chance-level performance was additionally examined at both the stimulus and art-education-group levels. For each of the six stimulus pairs, the observed number of correct classifications was compared with a binomial probability of 0.50 using two-sided exact binomial tests. Holm adjustment was applied across the six stimulus-level tests. In this task, a statistically significant accuracy below 0.50 indicates directional misclassification: participants selected the human-made image as AI-generated more often than the AI-generated image itself.

Within each of the three art-education groups, participant-level accuracy was also compared with 0.50 using two-sided one-sample *t*-tests. Holm adjustment was applied across these three group-specific chance tests. These within-group tests were used to determine whether the overall above-chance result was consistent across art-education backgrounds.

Differences among the three art-education groups were then evaluated using Welch’s one-way ANOVA because group sizes were unequal and Levene’s test indicated heterogeneity of variance. A significant omnibus result was followed by pairwise Welch *t*-tests with Holm adjustment.

Finally, differences among the six repeated binary items were examined using Cochran’s Q test, followed by Holm-adjusted pairwise McNemar tests. The exact binomial tests and Cochran’s Q/McNemar analyses address different questions: the former assess whether each stimulus produces performance above or below chance, whereas the latter assess whether accuracy differs across stimuli. For the self-reported cue-ranking task, ranking-retention status was compared across the three art-education groups using Pearson’s chi-square test, with Cramér’s *V* reported as the effect-size measure. As a sensitivity analysis, source-classification performance was compared between respondents with and without analyzable Q17 rankings. Item-level retained-versus-non-retained differences were examined using Pearson chi-square tests with Holm adjustment across the six stimulus pairs, whereas participant-level overall classification accuracy was compared using a Welch independent-samples *t*-test with Hedges’ g and a 95% confidence interval for the mean difference. Finally, the distributions of first-ranked and last-ranked cue categories were compared across art-education groups using Pearson chi-square tests with Cramér’s *V*.

### 3.5 Supplementary eye-tracking observation

Further details for this section are provided in the supplementary report (Supplementary Figure S3). A supplementary eye-tracking session was conducted in April 2025 at the Artificial Intelligence Laboratory of the School of Mechanical Engineering, Southeast University. Sixty participants who had completed the questionnaire took part in this supplementary experiment. The heat maps recorded the gaze data obtained when the participants, having already completed the discrimination task and understood the experimental procedure, viewed the familiar stimuli again. Among them, three art education groups each consisted of 20 participants. Gaze behavior was recorded using the D-Lab system with Dikablis eye-tracking glasses.

This session was designed as a supplementary observational component rather than as an independent eye-tracking experiment. The original research plan did not specify participant-level AOI-based inferential analysis as an outcome of the study. Consequently, participant-level fixation metrics were not retained in the final analytic dataset, and the archived material available for the present report consists of group-level heatmap exports. These heatmaps cannot be used to reconstruct fixation duration, fixation count, time-to-first-fixation, or participant-level viewing sequences. The heatmaps are therefore reported only as supplementary qualitative illustrations of aggregated gaze distribution.

Participants viewed the stimuli for 30 s, followed by a 10-s rest interval. Because trial-level questionnaire classification responses were not linked to the retained gaze material, no association between gaze location and classification accuracy is tested.

## 4 Results

### 4.1 Questionnaire data analysis

Art-education background was measured directly through the questionnaire and served as the grouping variable for the primary between-group comparisons. The original questionnaire item was: “What is your art-education background?” Participants selected one of three response categories: “no art background,” “some art background,” and “professional art background.”

The three categories were primarily defined according to whether participants had received formal and systematic art training and their disciplinary background. The no-art-background group comprised participants who had not received systematic art training and had not studied or received professional training in an art-related discipline. The some-art-background group comprised participants who had received some degree of art-related education or training but had not reached the level of professional disciplinary training. The professional-art-background group comprised participants who had received systematic and formal professional training in an art-related discipline. The final sample included 78 participants with no art background, 221 with some art background, and 57 with a professional art background. Among the 20 professional-art-background participants in the supplementary eye-tracking subsample, 10 were doctoral students in art-related disciplines at Southeast University.

The unequal group sizes reflect the actual composition of the questionnaire respondents rather than a predefined quota-based recruitment design. Because art-education background was the principal grouping variable, the observed group sizes were retained, and Welch’s ANOVA was used to accommodate unequal sample sizes and heterogeneity of variance across the three groups.

Regarding age distribution, the majority of participants were between 21 and 35 years old (*n* = 183, 51.40%), followed by those aged 10–20 years (*n* = 113, 31.74%) and those aged 36 years or older (*n* = 60, 16.85%; Table 1). The age composition varied descriptively across the three art-education groups. In the no-art-background group, participants were distributed relatively evenly between the 10–20-year (42.31%) and 21–35-year (41.03%) categories, with 16.67% aged 36 years or older. In the some-art-background group, more than half of the participants were aged 21–35 years (52.94%), while 34.39% were aged 10–20 years and 12.67% were aged 36 years or older. The professional art-background group showed a different age profile: 59.65% were aged 21–35 years, 33.33% were aged 36 years or older, and only 7.02% were aged 10–20 years. Overall, the professional art-background group contained a comparatively higher proportion of older participants, whereas the no-art-background group included a larger proportion of younger participants. Of the 356 questionnaire participants, 129 (36.2%) were male and 227 (63.8%) were female. The gender distribution was broadly similar across the three art-education groups: males accounted for 37.2% of the no-art-background group, 34.8% of the some-art-background group, and 40.4% of the professional art-background group. Regarding educational level, most participants were undergraduates (*n* = 246, 69.10%), followed by master’s students or degree holders (*n* = 76, 21.35%), doctoral participants (*n* = 24, 6.74%), and those with a high-school education (*n* = 10, 2.81%). The professional art-background group contained a comparatively higher proportion of postgraduate participants, whereas the no-art-background and some-art-background groups were predominantly composed of undergraduates. The three art-education groups were not evenly distributed in either sample size or demographic composition. In particular, the professional art-background group was relatively small (*n* = 57) and included a higher proportion of older and postgraduate participants. These differences reflect the naturally recruited sample rather than experimentally matched groups. Welch’s ANOVA was therefore used to accommodate unequal group sizes and variances, and the group comparisons should be interpreted with appropriate caution.

**Table 1:** Participant characteristics by art-education background.

Characteristic	No art background	Some art background	Professional art background	Total (*N* = 356)
Age group				
10–20 years	33 (42.31%)	76 (34.39%)	4 (7.02%)	113 (31.74%)
21–35 years	32 (41.03%)	117 (52.94%)	34 (59.65%)	183 (51.40%)
36 years and above	13 (16.67%)	28 (12.67%)	19 (33.33%)	60 (16.85%)
Total	78 (100%)	221 (100%)	57 (100%)	356 (100%)
Gender				
Male	29 (37.18%)	77 (34.84%)	23 (40.35%)	129 (36.24%)
Female	49 (62.82%)	144 (65.16%)	34 (59.65%)	227 (63.76%)
Total	78 (100%)	221 (100%)	57 (100%)	356 (100%)
Educational level				
High school	5 (6.41%)	5 (2.26%)	0 (0.00%)	10 (2.81%)
Undergraduate	64 (82.05%)	158 (71.49%)	24 (42.11%)	246 (69.10%)
Master’s	6 (7.69%)	48 (21.72%)	22 (38.60%)	76 (21.35%)
Doctoral	3 (3.85%)	10 (4.52%)	11 (19.30%)	24 (6.74%)
Total	78 (100%)	221 (100%)	57 (100%)	356 (100%)

#### 4.1.1 Overall and stimulus-level classification accuracy

Each participant completed six pairwise AI/human source-classification items. Participant-level accuracy was calculated as the proportion of pairs in which the AI-generated image was correctly identified, yielding a bounded score from 0 to 1. Across the 356 participants, mean classification accuracy was 0.549 (SD = 0.227, SE = 0.012). A two-sided one-sample *t*-test was conducted to determine whether participant-level accuracy differed from the 0.50 chance level expected under random responding in the two-alternative forced-choice task. Mean accuracy was significantly higher than 0.50, *t*(355) = 4.081, *p* < 0.001, mean difference = 0.049, 95% CI for the mean [0.525, 0.573], Cohen’s *d* = 0.216. Thus, participants showed a statistically reliable but small overall advantage over chance within the present six-pair stimulus set (Table 2).

**Table 2:** Statistics on the overall accuracy rate of the participants.

*N*	Range	Mean	Std. error	Std. deviation	Variance
356	0.000–1.000	0.54916	0.012045	0.227257	0.051646

The observed mean accuracy was significantly above the 0.50 chance level, *t*(355) = 4.081, *p* < 0.001, Cohen’s *d* = 0.216.

Classification accuracy was also calculated separately for each matched human–AI stimulus pair. Accuracy varied substantially across the six pairs, ranging from 32.6% for Pair 5 to 86.2% for Pair 1 (Table 3).

**Table 3:** Stimulus-level classification accuracy across the six matched human–AI pairs.

Pair	Valid *N*	Correct, *n*	Accuracy, % (95% CI)	Direction relative to 0.50	Holm-adjusted *p*
Pair 1	356	307	86.2 [82.3, 89.4]	Above chance	< 0.001
Pair 2	356	142	39.9 [34.9, 45.1]	Below chance	< 0.001
Pair 3	356	218	61.2 [56.1, 66.2]	Above chance	< 0.001
Pair 4	356	190	53.4 [48.2, 58.5]	No significant difference	0.223
Pair 5	356	116	32.6 [27.9, 37.6]	Below chance	< 0.001
Pair 6	356	200	56.2 [51.0, 61.2]	Above chance	0.045

Chance-level comparisons were conducted using two-sided exact binomial tests against *p* = 0.50. Reported *p* values are Holm-adjusted across the six stimulus-level tests.

To determine whether these values represented departures from random responding, each pair was compared with the 0.50 chance level using two-sided exact binomial tests with Holm adjustment.

Pair 1 (86.2%), Pair 3 (61.2%), and Pair 6 (56.2%) showed significantly above-chance classification accuracy (Holm-adjusted *p* < 0.001, *p* < 0.001, and *p* = 0.045, respectively), whereas Pair 4 (53.4%) did not differ significantly from chance (adjusted *p* = 0.223). Importantly, Pair 2 (39.9%) and Pair 5 (32.6%) were significantly below the 0.50 chance level (both adjusted *ps* < 0.001).

The below-chance results indicate systematic directional misclassification rather than simply greater task difficulty. For Pair 2, 214 of 356 participants (60.1%) selected the human-made painting as the AI-generated image; for Pair 5, 240 of 356 participants (67.4%) did so. Thus, the majority response in these two pairs was reversed relative to the documented image source. This pattern is consistent with the presence of pair-specific misleading visual or stylistic cues, although the present data do not identify which particular features produced the reversal.

A *post hoc* visual comparison of the highest- and lowest-performing stimulus pairs suggests several candidate sources of this item-level variation. Pair 1, which paired Frans Hals’s The Merry Drinker with its AI-generated counterpart, produced the highest classification accuracy (86.2%). Both images preserve the same half-length figure, large dark hat, ochre costume, and raised hand, but visible local differences remain in the rendering of the face and hand. Relative to the human-made source, the AI counterpart shows smoother and more regularized facial modeling, particularly around the eyes, mouth, and mustache, together with more blended articulation of the fingers in the raised hand. The contrast between Hals’s comparatively loose, irregular brushwork and the more smoothed local transitions of the AI counterpart may have provided participants with comparatively accessible source cues. This interpretation is descriptive, however, because the diagnostic contribution of these particular regions was not tested experimentally.

A different pattern is visible in Pair 5, Grant Wood’s American Gothic, which yielded the lowest accuracy (32.6%) and a reliable below-chance response pattern. The AI counterpart retains the original’s principal compositional organization extremely closely: the two figures occupy the same left–right positions, the pitchfork remains centrally aligned with the male figure, the Gothic-windowed house occupies the same background position, and the dominant palette and frontal geometry are largely preserved. The remaining differences are comparatively subtle and occur mainly in local facial modeling and surface texture. Moreover, the human-made original itself is characterized by highly controlled contours, smooth modeling, frontal rigidity, geometric organization, and limited gestural brushwork—visual properties that could plausibly overlap with lay expectations about algorithmically generated imagery. The fact that 67.4% of participants selected the human-made image as AI-generated is therefore consistent with a possible reversal of source cues: features of the original may have appeared more “AI-like” to many viewers than the corresponding AI image. The present data cannot determine whether this interpretation, prior familiarity, or another pair-specific feature caused the reversal.

Pair 2 provides a related case. The AI counterpart closely preserves Moroni’s full-length standing portrait, dark costume, curtain, tiled floor, and adjacent dog. Compared with the source painting, however, facial contours and several local details in the AI image appear somewhat smoother and more regularized, whereas the original retains painterly irregularities and unusual formal relations that may themselves appear artificial to contemporary viewers. With 60.1% of participants selecting the human-made painting as AI-generated, this pair again suggests that participants may sometimes have applied an “AI-like” visual heuristic to stylistic features already present in the historical original.

These comparisons indicate that stimulus-level variability involved more than a simple continuum of task difficulty. The easiest pair contained relatively visible local differences between a loosely painted historical source and its AI counterpart, whereas the two below-chance pairs combined high compositional similarity with characteristics of the human-made originals that could plausibly be misread as signs of AI generation. These observations identify candidate explanations for the direction of source judgments, but they do not establish which visual feature caused individual responses.

A Cochran’s *Q* test further showed that the probability of a correct response differed significantly across the six pairs, *Q*(5) = 264.682, *p* < 0.001. Holm-adjusted pairwise McNemar tests indicated that Pair 1 was identified more accurately than every other pair (all adjusted *ps* < 0.001). Pairs 2 and 5 produced lower accuracy than Pairs 3, 4, and 6; the difference between Pairs 2 and 5 was not significant after correction (adjusted *p* = 0.097). No significant differences were found among Pairs 3, 4, and 6 (all adjusted *ps* ≥ 0.164).

Taken together, the pair-level results reveal more than variation in task difficulty: different stimuli produced qualitatively different response patterns, including above-chance discrimination, chance-level responding, and systematic below-chance source attribution.

#### 4.1.2 Differences by art-education background

Participant-level accuracy was compared across the three art-education groups. The no-art-background group had a mean accuracy of 0.466 (SD = 0.191), the some-art-background group had a mean accuracy of 0.584 (SD = 0.228), and the professional art-background group had a mean accuracy of 0.526 (SD = 0.242).

Group-specific chance-level tests further clarified the pattern. The no-art-background group, despite having a numerical mean below 0.50 (*M* = 0.466, SD = 0.191), did not perform significantly below chance, *t*(77) = −1.584, *p* = 0.117, 95% CI [0.423, 0.509], Cohen’s *d* = −0.179. Its mean should therefore not be interpreted as evidence of systematic below-chance classification.

By contrast, the some-art-background group performed significantly above chance (*M* = 0.584, SD = 0.228), *t*(220) = 5.510, *p* < 0.001, 95% CI [0.554, 0.615], Cohen’s *d* = 0.371. The professional art-background group did not differ significantly from chance (*M* = 0.526, SD = 0.242), *t*(56) = 0.823, *p* = 0.414, 95% CI [0.462, 0.590], Cohen’s *d* = 0.109. Holm adjustment across the three group-specific tests did not alter these conclusions (Table 4).

**Table 4:** Accuracy statistics for three types of artistic backgrounds.

Art background	*N*	Mean	Std. deviation	Std. error
No art background	78	0.46581	0.190615	0.021583
Some art background	221	0.58446	0.227869	0.015328
Professional art background	57	0.52632	0.241501	0.031988
Total	356	0.54916	0.227257	0.012045

Group-specific one-sample tests against the 0.50 chance level showed significant above-chance performance only for the some-art-background group, *t*(220) = 5.510, *p* < 0.001. The no-art-background group, *t*(77) = −1.584, *p* = 0.117, and professional art-background group, *t*(56) = 0.823, *p* = 0.414, did not differ significantly from chance.

Thus, statistically reliable above-chance performance was observed only in the some-art-background group. The significant overall above-chance effect for the full sample should therefore not be interpreted as evidence that all three art-education groups independently discriminated image source above chance.

Levene’s test indicated that the homogeneity-of-variance assumption was violated, *F*(2, 353) = 3.690, *p* = 0.026. Group differences were therefore examined using Welch’s one-way ANOVA. The omnibus test showed a significant difference in classification accuracy across the three art-education groups, Welch’s *F*(2, 128.292) = 10.135, *p* < 0.001.

Holm-adjusted pairwise Welch tests showed that participants with no art background had lower accuracy than participants with some art background, *t*(160.016) = −4.482, adjusted *p* < 0.001, mean difference = −0.119, 95% CI [−0.171, −0.066]. The difference between the no-art-background and professional art-background groups was not significant after correction, *t*(103.060) = −1.568, adjusted *p* = 0.210, mean difference = −0.061, 95% CI [−0.137, 0.016]. The difference between the some-art-background and professional art-background groups was also not significant, *t*(83.549) = 1.639, adjusted *p* = 0.210, mean difference = 0.058, 95% CI [−0.012, 0.129] (Table 5).

**Table 5:** Welch’s ANOVA and Holm-adjusted pairwise comparisons by art-education background.

Test or comparison	Statistic	*df*	*p*	Mean difference	95% CI
Levene’s test	*F* = 3.690	2, 353	0.026	–	–
Welch’s ANOVA	*F* = 10.135	2, 128.292	<0.001	–	–
No art vs. some art	*t* = −4.482	160.016	<0.001	−0.11865	[−0.17093, −0.06637]
No art vs. professional	*t* = −1.568	103.060	0.210	−0.06050	[−0.13703, 0.01603]
Some art vs. professional	*t* = 1.639	83.549	0.210	0.05815	[−0.01239, 0.12869]

Pairwise *p* values were adjusted using the Holm procedure. Mean differences are calculated as the mean of the first group minus that of the second group.

The some-art-background group therefore showed the highest numerical mean, but the inferential results support only its difference from the no-art-background group. The professional group did not differ significantly from either of the other groups after adjustment for multiple comparisons. The pattern does not support a simple monotonic expertise advantage.

### 4.2 Self-reported visual judgment cues

#### 4.2.1 Task format and data selection

Question 17 assessed participants’ self-reported visual judgment cues. Participants were presented with four predefined categories of image details—facial details, hand details, clothing details, and background details—and were asked to rank these cues from highest to lowest according to their perceived reliance on them when judging whether a painting was AI-generated or human-made. Only responses providing a complete and unambiguous ordering of all four categories were considered analyzable. Responses were treated as excluded for this item when one or more categories were omitted, duplicated, or otherwise could not be coded as a unique first-to-last ranking. Of the 356 questionnaire participants, 245 (68.8%) provided valid complete rankings and were retained for the cue-ranking analysis.

Valid-ranking rates were 59.0% in the no-art-background group (46/78), 72.4% in the some-art-background group (160/221), and 68.4% in the professional art-background group (39/57). A Pearson chi-square test of independence showed that the association between art-education background and ranking-retention status did not reach statistical significance, *χ*^2^(2, *N* = 356) = 4.847, *p* = 0.089, Cramér’s *V* = 0.117. Although the no-art-background group showed a descriptively lower retention rate, there was no statistically reliable evidence that the probability of providing an analyzable ranking differed across the three art-education groups.

As a sensitivity analysis, source-classification performance was compared between the 245 respondents retained for the cue-ranking analysis and the 111 respondents without analyzable Q17 rankings. Item-level results are reported in Table 6. The retained and excluded counts were cross-checked against the full-sample stimulus totals reported in Table 3; for each of the six pairs, the two subgroup counts sum exactly to the corresponding full-sample number of correct classifications.

**Table 6:** Item-level source-classification accuracy by Q17 ranking status.

Pair	Retained *n*/*N* (%)	Excluded *n*/*N* (%)	Difference (pp)	*χ* ^2^	Raw *p*	Holm-adjusted *p*
Pair 1	215/245 (87.76%)	92/111 (82.88%)	4.87	1.528	0.216	0.866
Pair 2	102/245 (41.63%)	40/111 (36.04%)	5.60	0.998	0.318	0.866
Pair 3	143/245 (58.37%)	75/111 (67.57%)	−9.20	2.724	0.099	0.580
Pair 4	138/245 (56.33%)	52/111 (46.85%)	9.48	2.758	0.097	0.580
Pair 5	84/245 (34.29%)	32/111 (28.83%)	5.46	1.036	0.309	0.866
Pair 6	143/245 (58.37%)	57/111 (51.35%)	7.02	1.527	0.216	0.866

The two groups showed broadly similar directional patterns across the stimulus set. In particular, Pairs 2 and 5 remained comparatively difficult in both groups, and Pair 5 remained below the 0.50 benchmark in both the retained group (34.29%) and the excluded group (28.83%). Thus, partitioning the sample by Q17 ranking status does not alter the principal full-sample finding that Pair 5 produced a strong directional source-attribution reversal. Descriptive retained-versus-excluded differences ranged from −9.20 to +9.48 percentage points across the six pairs. These item-level values are reported primarily as a consistency and sensitivity check rather than as six additional inferential comparisons.

To examine whether cue-ranking retention was associated with stimulus-specific classification performance, we formally compared the retained and excluded groups for each of the six stimulus pairs using Pearson chi-square tests, with Holm adjustment across the six comparisons (Table 6). None of the six comparisons was statistically significant, either before or after Holm adjustment.

Particular attention was given to Pair 5 because it was one of the two stimuli showing significant below-chance source attribution in the full sample. Classification accuracy for Pair 5 was 34.29% (84/245) in the retained group and 28.83% (32/111) in the excluded group, a difference of 5.46 percentage points. The association between Q17 retention status and Pair 5 classification accuracy was not statistically significant, *χ*^2^(1, *N* = 356) = 1.036, *p* = 0.309, Cramér’s *V* = 0.054. The corresponding odds ratio was 1.29, 95% CI [0.79, 2.10].

Importantly, Pair 5 remained significantly below the 0.50 chance level within both subgroups: accuracy was 34.29% among respondents with analyzable Q17 rankings and 28.83% among those without analyzable rankings (both exact binomial *ps* < 0.001). Thus, the directional source-attribution reversal observed for Pair 5 was present in both the retained and excluded samples rather than being confined to one Q17-response subgroup.

At the participant level, overall source-classification accuracy was 0.561 (SD = 0.192) among respondents retained for the cue-ranking analysis and 0.523 (SD = 0.190) among respondents not retained (Table 7). The difference did not reach statistical significance, Welch’s *t*(214.62) = 1.742, *p* = 0.083, mean difference = 0.038, 95% CI [−0.005, 0.081], Hedges’ *g* = 0.198. Taken together with the stimulus-level analyses in Table 6, these results do not provide statistically reliable evidence that Q17 ranking retention was systematically associated with classification performance, either overall or for Pair 5 specifically. Nevertheless, because 31.2% of respondents did not provide analyzable rankings, the cue-ranking findings remain restricted to the retained subsample.

**Table 7:** Overall source-classification accuracy by Q17 ranking status.

Q17 status	N	M	SD
Retained	245	0.561	0.192
Excluded	111	0.523	0.190

Welch’s *t*(214.62) = 1.742, *p* = 0.083, mean difference = 0.038, 95% CI [−0.005, 0.081], Hedges’ *g* = 0.198.

Taken together, neither art-education background nor overall source-classification accuracy showed a statistically reliable association with cue-ranking retention. These analyses therefore provide no clear evidence that the retained cue-ranking sample was systematically selected on either dimension. Nevertheless, because 111 participants (31.2%) did not provide complete analyzable rankings, the subsequent cue-ranking findings are interpreted specifically as characterizing the 245 respondents who provided valid complete rankings rather than the full questionnaire cohort.

#### 4.2.2 Descriptive and inferential analysis of visual judgment cues

Within the retained cue-ranking sample (*n* = 245), the most frequently reported complete sequence was facial details → hand details → clothing details → background details (*n* = 66), followed by hand details → facial details → clothing details → background details (*n* = 47). These sequences represent participants’ retrospective reports of cue priority and should not be interpreted as objectively measured fixation sequences.

When the first-ranked cue was considered, facial details were most frequently ranked first (*n* = 115, 46.9%), followed by hand details (*n* = 76, 31.0%), background details (*n* = 32, 13.1%), and clothing details (*n* = 22, 9.0%). As shown in Table 8, facial details were the most frequently first-ranked cue in the some-art-background and no-art-background groups, whereas facial and hand details were equally frequent in the professional art-background group.

**Table 8:** Distribution of first-ranked visual judgment cues by art-education background.

Art-education background	Facial details	Hand details	Clothing details	Background details	Total
Professional art background	16 (41.0%)	16 (41.0%)	4 (10.3%)	3 (7.7%)	39
Some art background	78 (48.8%)	48 (30.0%)	15 (9.4%)	19 (11.9%)	160
No art background	21 (45.7%)	12 (26.1%)	3 (6.5%)	10 (21.7%)	46
Total	115 (46.9%)	76 (31.0%)	22 (9.0%)	32 (13.1%)	245

A Pearson chi-square test of independence showed that the distribution of first-ranked cue categories did not differ significantly across the three art-education groups, *χ*^2^(6, *N* = 245) = 6.185, *p* = 0.403, Cramér’s *V* = 0.112. Thus, although some descriptive variation was evident—for example, hand details were ranked first by 41.0% of the professional group compared with 26.1% of the no-art-background group—the overall distribution provided no statistical evidence of group-specific first-ranked cue priorities.

A similar pattern was observed for the last-ranked cue. Across the retained sample, background details were most frequently ranked last (*n* = 148, 60.4%), followed by clothing details (*n* = 46, 18.8%), hand details (*n* = 32, 13.1%), and facial details (*n* = 19, 7.8%). Table 9 shows that background details were the most frequently last-ranked category in all three art-education groups, ranging from 52.2% in the no-art-background group to 69.2% in the professional art-background group.

**Table 9:** Distribution of last-ranked visual judgment cues by art-education background.

Art-education background	Facial details	Hand details	Clothing details	Background details	Total
Professional art background	2 (5.1%)	3 (7.7%)	7 (17.9%)	27 (69.2%)	39
Some art background	13 (8.1%)	22 (13.8%)	28 (17.5%)	97 (60.6%)	160
No art background	4 (8.7%)	7 (15.2%)	11 (23.9%)	24 (52.2%)	46
Total	19 (7.8%)	32 (13.1%)	46 (18.8%)	148 (60.4%)	245

The distribution of last-ranked cue categories also did not differ significantly across art-education groups, *χ*^2^(6, *N* = 245) = 3.330, *p* = 0.766, Cramér’s *V* = 0.082. The small effect size indicates that between-group differences in last-ranked cue frequencies were limited.

Taken together, the cue-ranking results indicate a common descriptive tendency among respondents with valid rankings: facial and hand details were generally assigned greater reported priority, whereas background details were most frequently assigned the final position. However, neither the first-ranked nor the last-ranked cue distributions provided evidence of statistically distinct cue-prioritization patterns across the three art-education groups. These findings should be interpreted as retrospective self-reports of judgment strategy rather than as direct measures of visual attention, fixation sequence, or cue diagnosticity.

## 5 Discussion and conclusions

The present study found a modest overall advantage over chance in the six-pair source-classification task (*M* = 0.549), but the aggregate mean masks substantial and theoretically important heterogeneity. At the stimulus level, the results did not form a simple continuum from easier to more difficult items. Exact chance-level tests identified three distinct response patterns: above-chance classification for Pairs 1, 3, and 6, chance-level responding for Pair 4, and significantly below-chance classification for Pairs 2 and 5.

The below-chance results are particularly informative. In Pair 2, 60.1% of participants selected the human-made painting as AI-generated; in Pair 5, this proportion increased to 67.4%. These outcomes therefore reflect systematic reversal of source attribution rather than merely uncertainty or increased difficulty. A plausible interpretation is that particular features of these matched pairs consistently directed participants toward the incorrect source judgment. Such features might involve local structural details, stylistic coherence, perceived artificiality of the human-made work, or unusually convincing properties of the AI counterpart. The present design, however, does not permit identification of the responsible cue, and these possibilities should therefore be treated as hypotheses rather than established mechanisms.

This stimulus-specific reversal qualifies the interpretation of the overall above-chance mean. Source classification in the present task depended not only on the strength of discriminative information but also on its direction: some pairs supported correct source attribution, whereas others systematically favored the wrong attribution. This distinction is important because a below-chance item carries different psychological information from an item centered near 0.50. Chance-level performance is compatible with uncertainty or insufficient discriminative information; reliable below-chance performance instead indicates that the available information systematically favored the incorrect response. The art-education results reveal a second form of heterogeneity. Although the overall sample performed significantly above chance, group-specific tests showed that only the some-art-background group independently exceeded the 0.50 benchmark. The professional art-background group did not differ significantly from chance, and the no-art-background group also did not differ from chance despite its numerical mean of 0.466. The latter result is important because the raw mean alone could otherwise be mistaken for evidence of systematic below-chance performance.

These findings sharpen the interpretation of the Welch ANOVA. The significant group effect does not mean that the three groups occupied progressively different levels of a common above-chance ability. Rather, participants with some art background demonstrated reliable above-chance source classification, while the other two groups did not. At the same time, the professional group did not significantly differ from either group in the Holm-adjusted pairwise comparisons. The evidence therefore remains inconsistent with a simple monotonic expertise hierarchy. This pattern can be considered alongside previous research on AI/human artwork discrimination. Grassini and Koivisto (2024), for example, reported substantial difficulty in distinguishing AI-generated from human-made artworks within their stimulus set, whereas van Hees et al. (2025) found statistically above-chance discrimination in a paired-artwork paradigm, although the effect was also relatively small. The present result is therefore broadly compatible with evidence that viewers can sometimes discriminate image source above chance while nevertheless showing considerable uncertainty. Differences across studies should be interpreted cautiously because stimulus sets, generative models, presentation formats, and classification procedures vary substantially. The marked variation across the six stimulus pairs in the present study further suggests that the perceptual information available for source classification was unevenly distributed across images. This interpretation can be situated within recent high-level work on the perceptual organization of visual art. Iigaya et al. (2021) demonstrated in Nature Human Behavior that esthetic responses to visual artworks can be predicted from a combination of low-level visual properties and higher-level semantic features, rather than from a single class of pictorial information. Their subsequent neuroimaging study showed that low- and high-level features relevant to esthetic valuation are represented hierarchically across the visual system and integrated into higher-order value computations (Iigaya et al., 2023). Although these studies addressed esthetic valuation rather than AI-source classification, they provide an important mechanistic framework for interpreting the present stimulus-level variation: viewers’ judgments are unlikely to depend on a single local anomaly and may instead reflect the integration of multiple levels of pictorial information. Recent psychophysical evidence reinforces this point. Boger and Firestone (2025) showed that artistic style itself supports systematic perceptual tuning and can be perceptually represented partly independently of depicted content. Accordingly, differences among the six pairs may have arisen from varying combinations of local structural details, global compositional relations, and stylistic coherence. The present data do not permit these contributions to be separated, but the large pair-level differences indicate that they should be examined directly in future work.

The art-education findings also argue against a simple expertise hierarchy. Participants with some art background showed the highest mean accuracy and performed significantly better than participants with no art background, whereas the professional art-background group did not significantly outperform either group after multiplicity correction. This pattern is broadly consistent with van Hees et al. (2025), who found no statistically significant relationship between art experience and AI-detection accuracy in their paired-artwork task. It also fits a broader account of esthetic cognition in which prior experience modulates perceptual and evaluative processing without determining a single optimal response. Nadal and Skov (2025), in their Nature Reviews Psychology account of esthetic experience, argue that sensory evaluation is jointly shaped by properties of the stimulus and by learned experience, semantic regulation, and the observer’s current state. From this perspective, formal art training may alter the weighting and interpretation of visual information without necessarily producing monotonically higher source-classification accuracy.

The present non-linear pattern can therefore be interpreted more cautiously as evidence that art-related experience and source-classification performance are not interchangeable constructs. Professional training may expand the range of stylistic, compositional, historical, or material criteria available to a viewer, but a larger repertoire of criteria need not yield higher performance in a binary source-classification task. This distinction is especially relevant when the stimuli are deliberately matched in broad subject matter, composition, and style. The present results consequently neither demonstrate an expert disadvantage nor support a simple expert advantage. They instead identify a mismatch between formal expertise level and classification performance that warrants targeted investigation. Future studies could test this directly by recording trial-level reasons for each judgment and examining whether art-education groups assign different weights to local anomalies, stylistic consistency, composition, brushwork simulation, or prior familiarity.

The face- and hand-related observations can likewise be situated more precisely within contemporary visual-attention research. The recurrent concentration of gaze around faces in the present figure-based heatmaps is consistent with a substantial literature showing that faces occupy a privileged position in visual selection. De Haas et al. (2019), analysing eye movements from more than 100 observers viewing complex scenes, found large and stable individual differences in semantic visual salience, including the tendency to fixate faces; importantly, individual face salience was associated with face-recognition ability. More recently, Kollenda et al. (2026) reported in Nature Human Behavior that individuals differ reliably in their propensity to fixate faces and that this gaze tendency is related to category-selective neural representations in the ventral visual stream and to face-recognition performance. These findings provide a stronger empirical basis for interpreting recurrent facial gaze concentration as part of a general property of active visual selection rather than as a cue unique to AI-generated art. The corresponding group-level heatmaps are provided in Supplementary Figure S3.

Among respondents who provided valid complete rankings, facial and hand details were frequently assigned early positions, whereas background details were most often placed last. However, inferential comparisons showed no significant differences in the distributions of either first-ranked or last-ranked cues across art-education groups. Thus, the self-report data do not support the claim that the three groups adopted categorically different visual cue strategies. Rather, they indicate a broadly shared reported emphasis on figure-related details, particularly faces and hands. This descriptive pattern can be situated alongside previous research showing that faces often attract early attention during the viewing of figurative paintings and that attention to faces and action-related body regions varies with pictorial context (Massaro et al., 2012; Villani et al., 2015). The present findings, however, concern retrospective cue rankings rather than objective gaze sequence and therefore should not be treated as direct evidence of visual-attention mechanisms. A further implication concerns the relation between local and global pictorial information. Participants frequently reported faces and hands as relevant judgment cues, yet the substantial stimulus-level variation suggests that these local regions alone cannot account for classification performance. The hierarchical feature-integration framework developed by Iigaya et al. (2021, 2023) and the psychophysical evidence for style perception reported by Boger and Firestone (2025) together suggest a more plausible interpretation: source judgments may emerge from interactions among local structural information, global compositional organization, stylistic regularities, and higher-level expectations. This interpretation also helps explain why additional formal training did not translate into a simple performance gradient. Different observers may attend to or weight these information sources differently, and greater expertise may change the structure of the judgment process without guaranteeing a more accurate binary decision. This remains an interpretation rather than a mechanism demonstrated by the current data.

Several limitations constrain these conclusions. The stimulus set contained only six matched pairs and was heterogeneous in genre, figure configuration, and compositional structure. The wide variation in stimulus-level accuracy indicates that the overall mean should not be treated as a uniform property of AI-generated painting. This study did not document the sampler, the number of sampling steps, the CFG coefficients, the resolution, or the image-to-image (img2img) denoising strength; therefore, this set of stimulus materials cannot be reproduced. Familiarity with the museum source paintings was not measured at the item level and may have influenced classification for some participants. This limitation is particularly relevant given evidence that face-directed gaze varies systematically across individuals and is associated with perceptual and neural characteristics (de Haas et al., 2019; Kollenda et al., 2026).

Future studies should use larger and more balanced stimulus sets, incorporate multiple image-generation models, and measure prior familiarity with individual source paintings. Eye-tracking studies should preregister semantically defined AOIs, retain participant-level gaze records, and collect source-classification judgments on each trial. Such designs would permit mixed-effects analyses of both participant- and stimulus-level variation and enable direct tests of whether attention to particular image regions predicts correct classification. Combining these measures with computational estimates of low-level image statistics, global composition, and stylistic similarity would further clarify the relative contribution of local and global visual information.

In conclusion, the present study shows that AI/human source classification within matched painting pairs cannot be summarized adequately by a single overall accuracy value. Although aggregate accuracy was modestly above chance, the stimulus-level results ranged from reliable above-chance discrimination to systematic below-chance misclassification. In two pairs, most participants selected the human-made painting as AI-generated, indicating a directional source-attribution bias rather than merely greater task difficulty. The art-education results were similarly heterogeneous. Only the some-art-background group performed significantly above chance, whereas the no-art-background and professional groups did not differ reliably from 0.50. Together with the non-linear between-group differences, this pattern indicates that formal art education is not associated with a simple monotonic increase in source-classification performance. The principal contribution of the study therefore lies in identifying stimulus-specific directionality in source attribution, non-linear differences associated with art education, and variation in reported judgment cues. Future work should determine which visual and contextual features produce accurate versus systematically reversed source judgments.

## Data Availability

The datasets presented in this article are not readily available because the questionnaire dataset includes responses from 14 participants younger than 18 years. Under the approved ethics protocol, public disclosure of data involving minors requires additional consent from their legal guardians. Because such consent for public data sharing was not obtained, the participant-level raw questionnaire dataset is not publicly available. De-identified aggregate data supporting the reported analyses are presented in the article and Supplementary material. The original participant-level eye-tracking records are no longer available; only the archived group-level heatmap exports described in the manuscript were retained. Requests to access the datasets should be directed at huangyilx@gmail.com.
