# Supplementary material for: Judging AI-generated paintings: art-education differences in source classification
Source: Front Psychol. 2026 Oct 1;17:1903106. doi: 10.3389/fpsyg.2026.1903106 (PMC13671107; doi:10.3389/fpsyg.2026.1903106)
Supplement: Supplementary file 1 [file Data_Sheet_1.PDF]

Supplementary Material

1 Supplementary Information

1.1 Experimental Equipment and Presentation Methods

The visual stimuli were presented on a 60-inch monitor with a resolution of 1920 x 1080 pixels. The experiment was conducted in a sound-attenuated laboratory to reduce the influence of external noise on participants' attention. The experiment was conducted in a controlled laboratory environment to minimize external distraction. Gaze behaviour was recorded using the D-Lab system developed by Ergoneers together with Dikablis eye-tracking glasses. For the present study, the retained eye-tracking outputs consisted of aggregated heatmap visualizations rather than participant-level fixation datasets suitable for retrospective AOI-based statistical analysis. The eye-tracking results are therefore reported qualitatively as spatial summaries of gaze concentration. No fixation-duration, fixation-count, time-to-first-fixation, or AOI-level inferential statistics are reported.

1.2 Procedure

To approximate the viewing conditions of an art gallery, participants viewed the images while standing rather than sitting. Wearing Dikablis eye-tracking glasses, they viewed the AI-generated and human-made paintings from a distance of 100 cm from the screen, with a fixed tabletop placed in front of the body for support. The experiment began with the eye-calibration procedure provided by D-Lab . Each set of works was viewed for 30 seconds, followed by a 10-second rest period to reduce eye fatigue. Because the retained eye-tracking material did not permit participant-level linkage between gaze behaviour and trial-level source-classification responses, the heatmaps were used only as exploratory visualizations of aggregated gaze distribution. They were not used to estimate classification accuracy, identify predictive gaze features, or test differences in AOI-level attention between participant groups or image-source conditions.

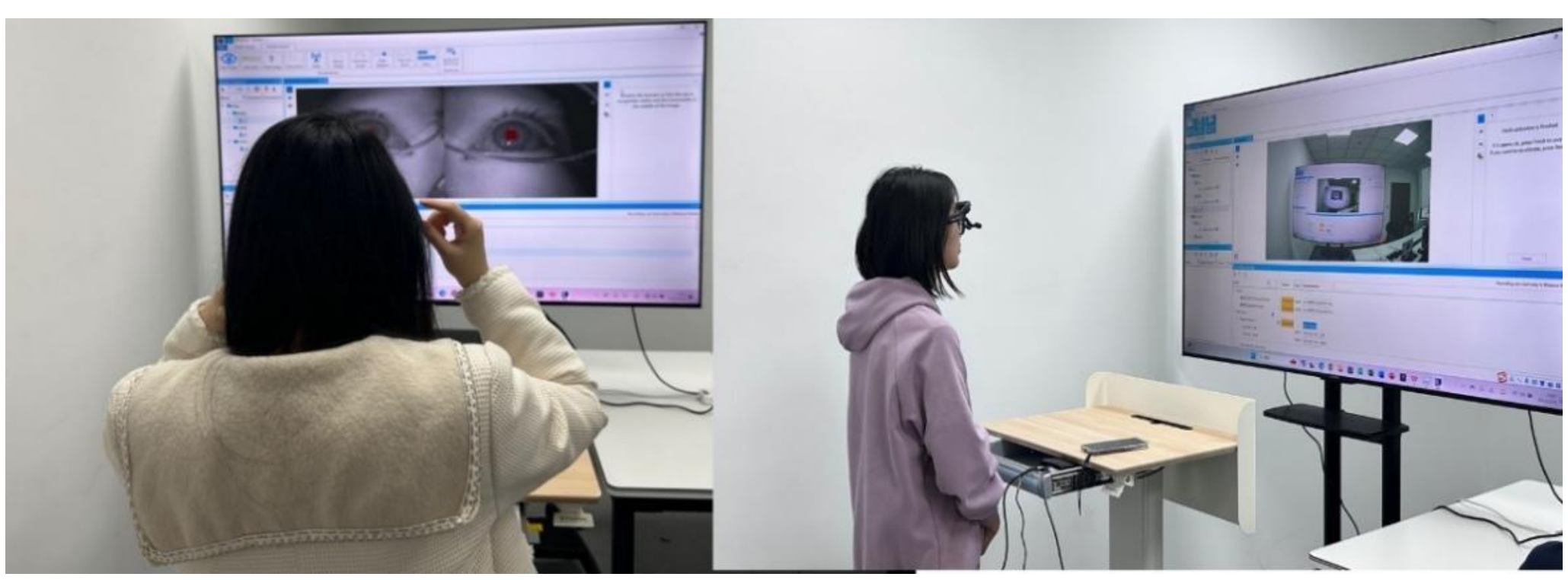

Supplementary Figure S1 Cross calibration before experimentation

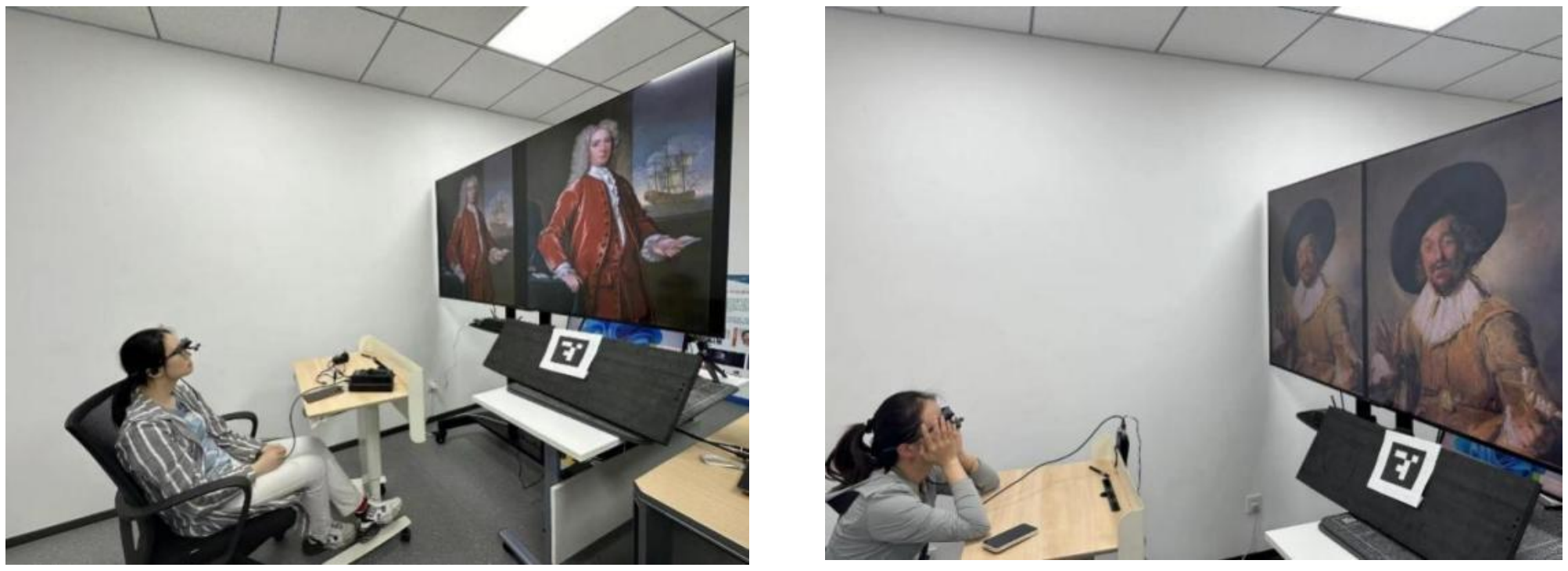

Supplementary Figure S2 Experimental process and environment

1.3 Prompts for Generating AI Art

Fig1：oil painting, by Frans Hals, Dutch Golden Age baroque portrait, half body shot, a merry middle-aged Dutch man with a tipsy smile, looking directly at viewer, curly brown hair, mustache and goatee, wide black felt hat, tan wool jacket, white lace ruff collar, black leather belt with gold buckle, holding a glass beer cup in left hand, right hand raised in gesture, relaxed posture loose bold brushstrokes, visible impasto texture, expressive painterly style, warm side lighting, soft chiaroscuro, warm brown color palette, plain dark background, classic canvas texture, subtle aged patina.

Fig2：full-length standing portrait of a middle-aged man with short dark brown curly hair and a full beard, solemn and steady in demeanor, gazing directly at the viewer, standing with a slight body turn. His right hand holds brown leather gloves, and his left hand hangs naturally at his side. He wears a heavy long black velvet robe over black inner garments, with a narrow white standing collar peeking out at the neckline and white lining visible at the cuffs; the velvet fabric displays natural, heavy draped folds. A dark green velvet curtain hangs on the left side of the frame, trimmed with delicate gold embroidered lace. The background features light gray stone pilaster walls, and the floor is paved with checkered marble tiles in orange, off-white, and olive green. Soft side lighting illuminates the scene from the upper left, with delicate, natural chiaroscuro transitions. Rendered in classical realist oil technique on canvas, with subtle, substantial brushwork and an overall solemn, restrained color palette. Symmetrical, dignified vertical full-length portrait composition, presented as a museum-grade high-resolution oil painting scan with authentic linen canvas texture and fine detail.

Fig3：An aesthetic portrait—a stunning full-length portrait in portrait orientation—features a calm and refined middle-aged gentleman looking directly at the viewer with a restrained and serene expression. He wears a long, dark brown wrap-style coat with a flowing, slender silhouette, layered over a dark suit, a vest, and a white shirt with a stand-up collar. His right hand hangs naturally at his side, holding a black bowler hat. The background features a minimalist, unified warm brown-beige palette, with the floor and background wall blending seamlessly into one another, free of any superfluous decorative elements. The low-saturation, uniform color scheme minimizes contrast between light and shadow, while soft, blurred edges and a flat, brushstroke-blended finish create an overall serene and elegant atmosphere. The painting possesses the matte, classical texture of an oil painting on canvas, with a delicate canvas grain.

Fig4：An 18th-century Neoclassical oil painting depicts an elegant and noble woman wearing a flowing pale pink gown with a light blue sash and white long sleeves. Standing on the left side of the painting, she extends her right hand to touch a golden disc placed on an ornate golden tripod adorned

with garlands. Seated to her right is a young girl wearing a green dress. The background features a classical temple-style architecture with stone columns and marble steps. In the upper left corner of the background stands a white marble sculpture of the "Three Graces." Behind the tripod sits a large bronze urn. The painting employs soft yet dramatic lighting and shadow effects, utilizing chiaroscuro techniques. It is rendered in soft pastel tones, blending Romantic and Academic styles, and is exceptionally rich in detail.

Fig5: Oil painting, a frontal double portrait: an elderly farming couple with solemn expressions stands in the foreground. The balding man wears thin-framed round glasses and has a stern, rigid expression; he is dressed in a black jacket, a striped shirt, and blue denim work pants, and holds a pitchfork upright in his right hand. The woman beside him has blonde hair parted down the middle and a composed expression. She wears a dark brown polka-dot dress with a white Peter Pan collar and a gold relief brooch pinned to her chest. Background: A white Gothic Revival-style farmhouse with arched windows; to the left is a red barn, rounded green trees, and a pale gray-blue sky. The composition is symmetrical, with flat modeling, smooth matte brushstrokes, clear and sharp contours, subdued earth tones, a vintage American rural aesthetic, and visible canvas texture.

Fig6：A classical oil painting in a realistic style, depicting a three-quarter-length portrait of a dignified and composed middle-aged gentleman. He wears a long, grayish-white powdered wig and has a solemn expression as he looks directly at the viewer. He is dressed in a long, brick-red velvet tailcoat with a dense row of fabric buttons of the same color down the front, worn over a white shirt with white ruffled trim at the neckline, and white lace ruffles at the cuffs. His right hand rests on the edge of a table covered with a deep green velvet tablecloth, upon which papers and documents are scattered; his left hand holds a folded sheet of letter paper. His posture is composed and dignified. The background features a dark, deep-brown interior; through the window on the right, one can see the twilight sea and a large sailing merchant ship against a gray-blue, overcast sky.

1.4 Exploratory Qualitative Description of Eye-Tracking Heatmaps

The eye-tracking component is reported as a qualitative description of archived group-level heatmaps. Because participant-level gaze records were not available for retrospective AOI-based analysis, the heatmaps are not used to estimate fixation duration, fixation count, time to first fixation, or statistically test differences between art-education groups or between AI-generated and human-made stimuli. Accordingly, color intensity in the heatmaps is interpreted only as a descriptive indication of relatively concentrated aggregated gaze within a displayed image. To account for the fatigue that might result from prolonged viewing, we selected four images as stimuli for the supplementary eye-tracking observation.

Across the figure-based stimuli shown in the heatmap visualizations, relatively concentrated gaze was repeatedly visible around facial regions in all three art-education groups. This pattern was particularly apparent in portraits and double-figure compositions, in which the strongest heatmap concentrations were commonly located around one or more faces. In several stimuli, additional localized concentrations were also visible around hands and other figure-related regions. These secondary concentrations appeared in more than one participant group, although the present data do not permit statistical comparison of their magnitude or frequency across groups.

The heatmaps also suggest that gaze distribution outside the principal figure regions was generally more spatially diffuse in the selected examples. However, this observation should not be interpreted as evidence that background regions received significantly less attention, because differences in AOI size,

fixation duration, fixation frequency, and viewing sequence cannot be quantified from the retained heatmap visualizations.

These heatmap patterns can be considered alongside the self-reported cue rankings in Section 4.2: facial and hand details were frequently ranked as salient cues, and visible gaze concentrations occurred around corresponding figure regions in some stimuli. As noted above, the heatmaps provide exploratory visual context only and are not used to infer fixation order, cue diagnosticity, or group differences in gaze strategy. They are retained primarily to inform hypotheses for future AOI-based studies.

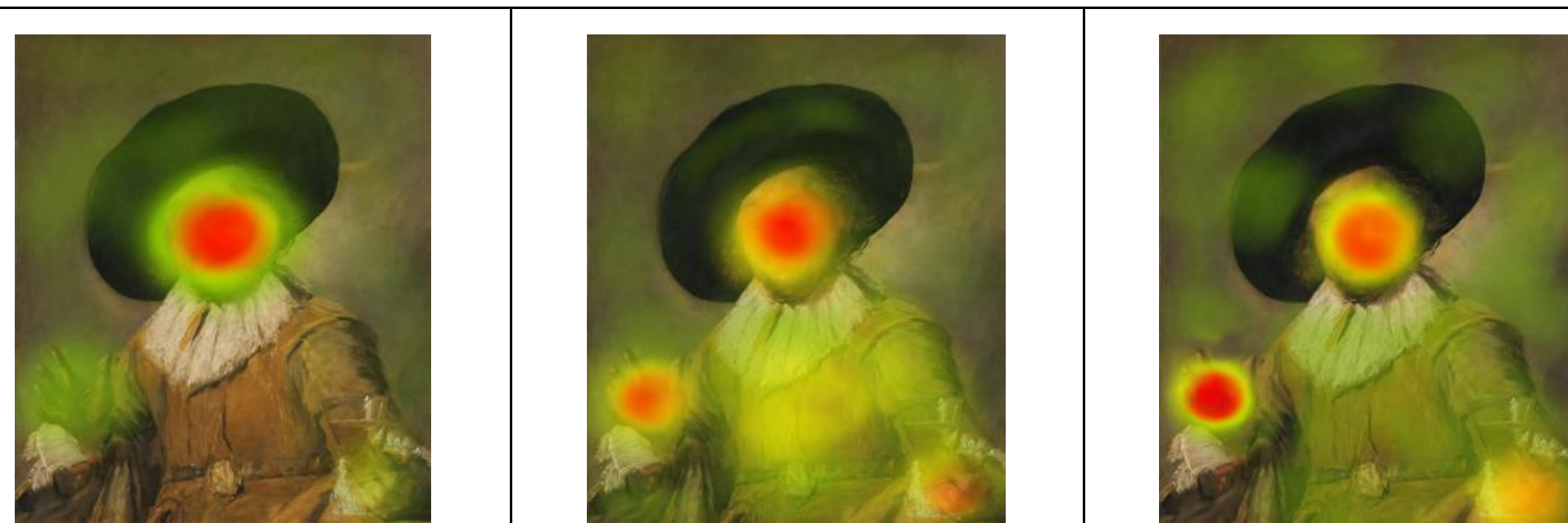

AI Art 01

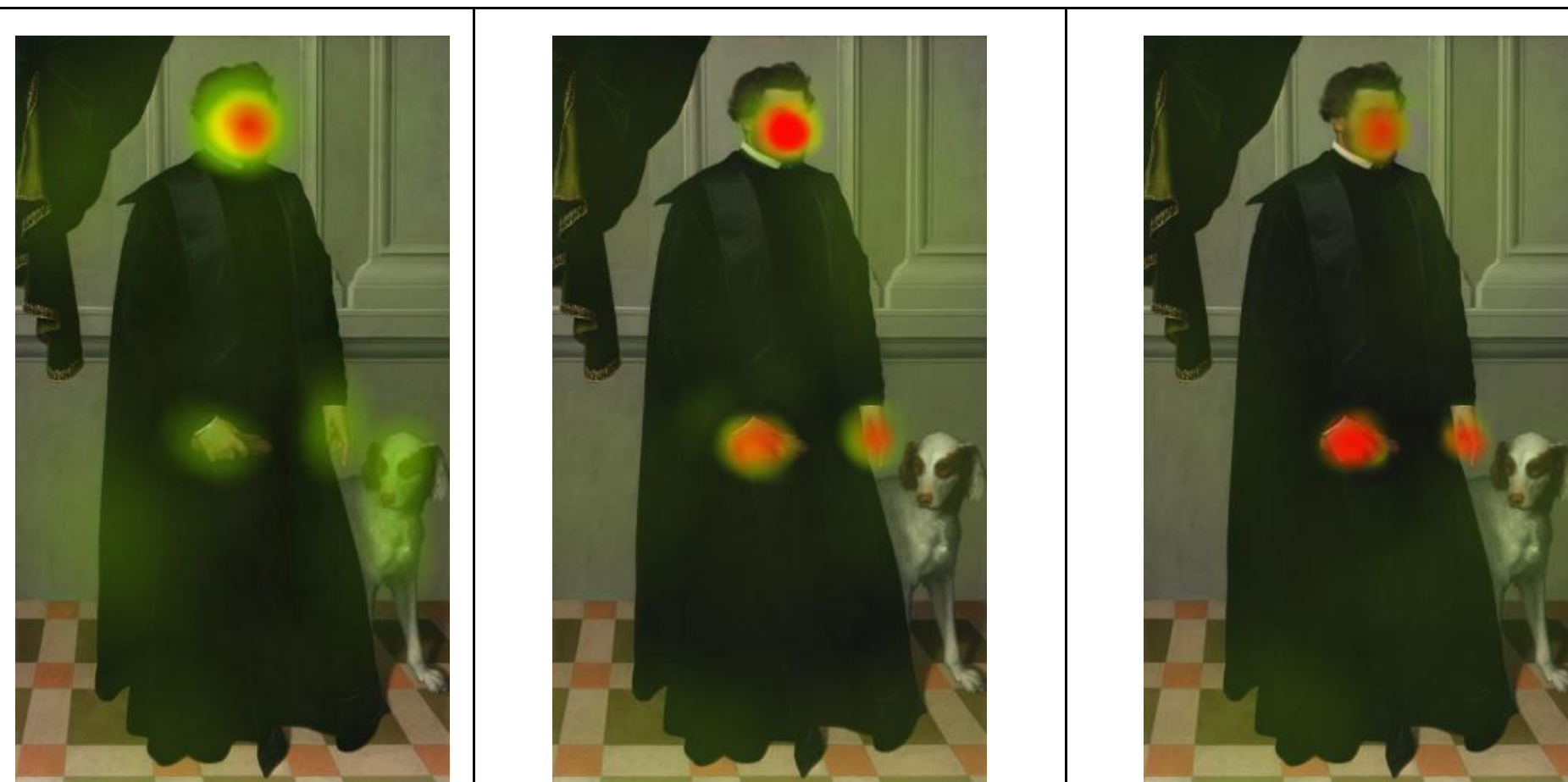

AI Art 02

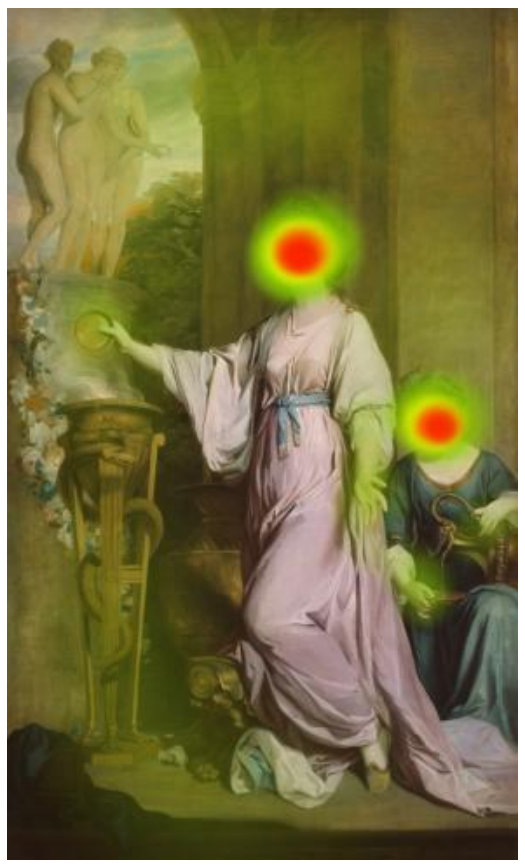	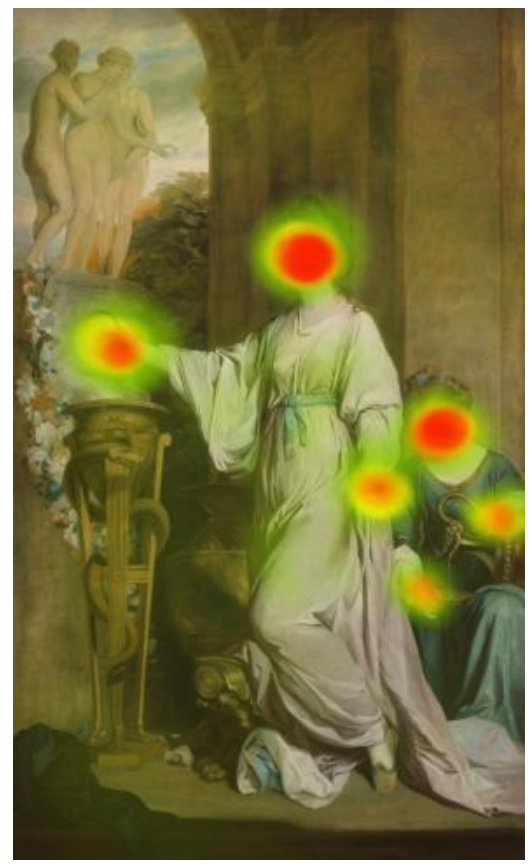	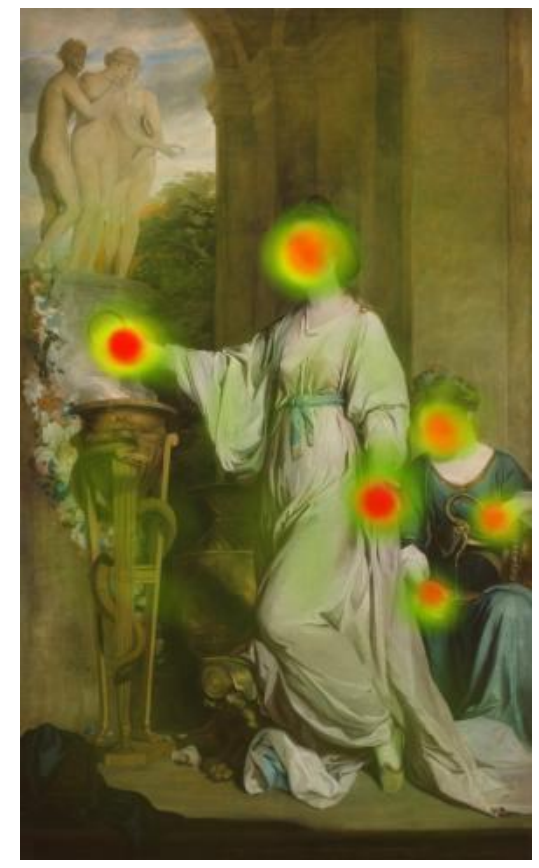
AI Art 04		
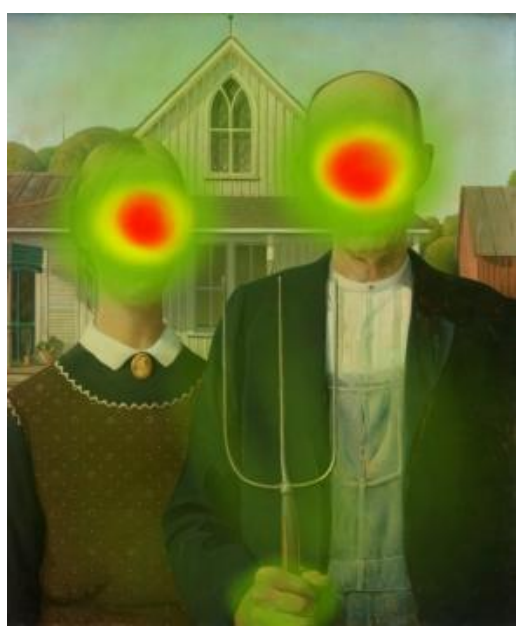	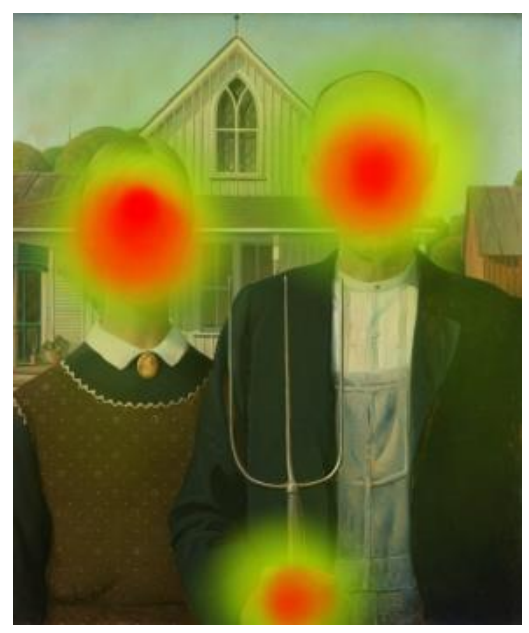	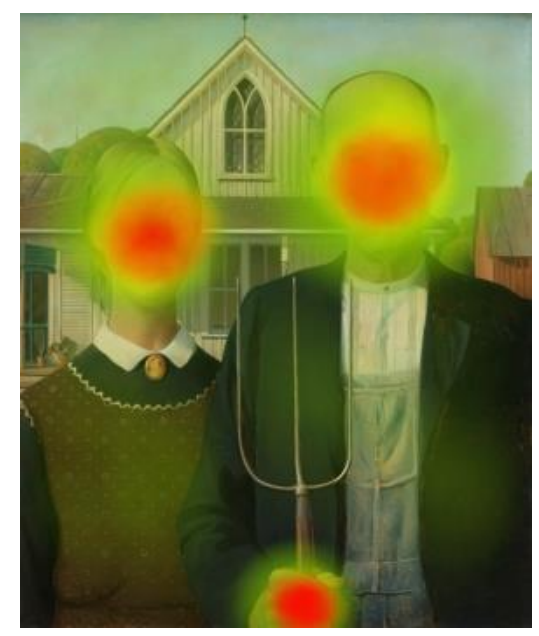
AI Art 05		
No art background	Some art background	Professional art background
Supplementary Figure S3. eye-tracking heatmaps		
